# The Efficacy of Parent Management Training With or Without Involving the Child in the Treatment Among Children with Clinical Levels of Disruptive Behavior: A Meta-analysis

**DOI:** 10.1007/s10578-022-01367-y

**Published:** 2022-07-05

**Authors:** Maria Helander, Martin Asperholm, Dan Wetterborg, Lars-Göran Öst, Clara Hellner, Agneta Herlitz, Pia Enebrink

**Affiliations:** 1 Division of Psychology, Department of Clinical Neuroscience, Karolinska Institutet, Solna, Sweden; 2 Department of Psychology, Stockholm University, Stockholm, Sweden; 3 Centre for Psychiatry Research, Department of Clinical Neuroscience, Karolinska Institutet & Stockholm Health Care Services, Stockholm County Council, Stockholm, Sweden

**Keywords:** Meta-analysis, Parent Management Training (PMT), Disruptive behavior disorder, Randomized controlled trials, Parent–Child Interaction Therapy (PCIT), Cognitive behavioral therapy (CBT)

## Abstract

A systematic review and meta-analysis was conducted where we evaluated the effects of Parent Management Training (PMT), Parent–Child Interaction Therapy (PCIT) and PMT combined with child cognitive behavioral therapy (CBT) using data from 25 RCTs on children with clinical levels of disruptive behavior (age range 2–13 years). Results showed that PMT (*g* = 0.64 [95% CI 0.42, 0.86]) and PCIT (*g* = 1.22 [95% CI 0.75, 1.69]) were more effective than waiting-list (WL) in reducing parent-rated disruptive behavior, and PMT also in improving parental skills (*g* = 0.83 [95% CI 0.67, 0.98]) and child social skills (*g* = 0.49 [95% CI 0.30, 0.68]). PCIT versus WL had larger effects in reducing disruptive behavior than PMT versus WL. In the few studies found, the addition of child CBT to PMT did not yield larger effects than PMT or WL. These results support offering PMT to children with clinical levels of disruptive behavior and highlight the additional benefits of PCIT for younger ages.

**Supplementary Information:**

The online version contains supplementary material available at 10.1007/s10578-022-01367-y.

Disruptive behavior disorders (DBD), such as oppositional defiant disorder (ODD) [1] and conduct disorder (CD) [1] are strenuous conditions for children and families, associated with a higher risk for antisocial development [2] and internalizing disorders [3]. Disruptive behavior disorders are also associated with a substantial burden and high costs for society [4–7]. Here, we investigate the effectiveness of three therapy programs in the treatment of disruptive behavior disorders and compare their relative effectiveness.

Previous research has shown that Parent Management Training (PMT) is an effective treatment for disruptive behavior during childhood. In PMT, parents are taught strategies for handling behavior problems and improving the quality of the parent–child relationship. PMT programs embrace positive parental involvement with the child, increased parental attention on adaptive behaviors, and enhanced parent–child communication. PMT also includes teaching parents to prepare instructions for the child ahead of time, to use clear instructions, to respond with positive attention and warmth especially when the child shows desirable behavior, and to reduce the risk of reinforcing negative behavior by not focusing on minor disruptive behavior and work with non-punitive consequences [8].

The effects of PMT compared to waiting-list (WL) or treatment as usual (TAU) have been examined in an extensive number of clinical trials and in several meta-analyses and reviews [e.g., 9–18], showing moderate between-group effect sizes in reduced ODD- and CD-symptoms, or disruptive behavior in general. However, when examining the effects of PMT in randomized controlled trials, few meta-analyses focus solely on the effects of PMT for children with disruptive behavior within a clinical range (i.e., children with disruptive behavior diagnosis or disruptive behavior problems above a clinical cut-off). The effects of PMT on children with large problems have been captured in two earlier meta-analyses by Leijten and colleagues by including studies conducted in treatment settings [17, 19]. However, an inspection of the studies included in these meta-analyses, indicates that although they are conducted within treatment settings, a proportion of these include children with sub-clinical levels of disruptive behaviors, or focus on attention deficit/hyperactivity disorder (ADHD). Another example of a meta-analysis with studies on children with ADHD alongside studies on children with disruptive behavior disorders is Battagliese et al. [14].

We have only found three meta-analyses that exclusively included randomized controlled trials (RCT) in children and adolescents with clinical levels of disruptive behaviors. In 2005, Bradley and Mandell conducted a meta-analysis on studies of school-aged children, with five studies on children with ODD and two studies on aggressive behavior [15]. In that meta-analysis, PMT was evaluated alongside child directed treatment and school-based treatment, compared to any control, demonstrating reduced disruptive behavior of PMT (standardized mean difference [*SMD*] = 1.06, 95% CI 0.70 to 1.41) as well as of child directed treatments (*SMD* = 0.93, 95% CI 0.52 to 1.34) on disruptive behavior outcomes. Only seven studies were included in this meta-analysis, conducted several years ago, and the studies on aggressive behavior were not above a clinical cut-off. Furlong and colleagues [13] included studies of PMT for families with children in the clinical range. The mean effect size reported was 0.53 (95% CI 0.34 to 0.72). This meta-analysis included studies up until 2010. A third meta-analysis based on RCTs [20], included 17 studies of children and adolescents 2–18 years of age with an ODD- or CD diagnosis or clinical levels of conduct disorder symptoms. This meta-analysis included PMT and other psychosocial treatments for ODD and CD, such as school-based treatments and multi-systemic treatments for youth, thereby complicating the possibility to draw conclusions regarding PMT effectiveness specifically.

To summarize, previous PMT meta-analyses that include RCTs and have samples with clinical levels of disruptive behavior in children are few and have either not focused solely on PMT efficacy [20], were performed over a decade ago [13, 15], have included studies on children with ADHD only among the children with disruptive behavior disorders [14] or, in addition to studies with clinical samples also included studies on children whose disruptive behavior problems were not above a clinical cut-off, even though referred to a treatment setting [17, 19]. Although earlier meta-analyses have contributed with important information regarding mixed samples and it can be assumed that PMT has a similar effect on children with clinical levels of disruptive behavior, it has not been investigated in a separate meta-analysis on PMT.

The possible long-term effects of PMT on child disruptive behavior have been evaluated in a meta-analysis by Van Aar et al. [12]. The authors included children with clinical as well as non-clinical disruptive behaviors and identified a sustained effect of parenting interventions, regardless of the initial levels of child disruptive behavior problems, age, gender or ethnicity. Long-term effects on clinical levels of disruptive behavior have also been examined in a meta-analysis, where PMT and other types of treatment modalities (such as child CBT alone, PMT combined with child-directed CBT, and multidimensional treatments such as Multisystemic treatment) were evaluated with no comparison or compared to WL [10]. Long-term within-group effects were examined from post-treatment to follow-up, showing sustained treatment effects on conduct problem outcomes. A limitation with the meta-analysis by Fossum et al. [10], was the inclusion of non-RCTs and the inclusion of different treatment modalities alongside PMT in the analysis, making the specific long-term effects of PMT hard to distinguish.

PMT delivered to parents individually or in groups is often the recommended treatment of choice in clinical guidelines [e.g., 21]. Another path to decreased disruptive behavior is to include or address the child in the treatment. In the NICE guidelines, two treatment approaches where the child is involved are described: (1) individual parent and child training programs, where the parent uses principles learned in treatment with the child, and receives guidance and feedback from the therapist (e.g., as in Parent–Child Interaction Therapy [PCIT]) [22], and (2) child focused social- and cognitive problem-solving and social skills training programs where the child takes part in the treatment by itself (e.g., Cognitive Behavioral Therapy [child CBT]). PCIT [22] is an individual parent and child training program (ages 2–7 years) where the therapist guides the parent via a bug-in-the-ear device with the child present in the treatment room in order to coach the parent to enhance the parent–child relationship, improve parenting skills, and to reduce the child’s externalizing behavior problems. PCIT has shown reduced behavior problems in meta-analyses on clinical and subclinical levels of disruptive behavior and non-RCTs [23–25], but no meta-analysis evaluating the effects of PCIT with both RCTs and clinical levels of disruptive behavior as inclusion criteria has yet been conducted. Child CBT involves social and cognitive problem-solving training for children 9–14 years of age [21]. In child CBT, children with disruptive behavior are taught strategies to handle aggression, regulate emotions, use problem-solving techniques, and practice perspective-taking. A recent meta-analysis examined the effects of child social skills training on aggression, delinquency, and violence in either universal, selective, or indicated prevention studies, showing a medium effect size post-treatment for indicated samples in moderator analyses (*d* = 0.49, 95% CI 0.36–0.62) [26]. Other studies have evaluated child CBT in combination with PMT and reported an increased effect size compared to when only PMT is delivered [27] or compared to a control group at 1-year follow-up [28], although not all studies have reported such effects [29]. PMT with child CBT is often studied with an addition of kindergarten- or school-based treatment, where teachers are involved in the treatment [30, 31]. In previous meta-analyses, the addition of school-based treatment has sometimes been incorporated in the calculation of effect sizes [20, 32]. Thus, for clinicians and policymakers, it would be important to synthesize the potential additive effect of child inclusion in or alongside the PMT treatment at clinical levels of disruptive behavior as well as without a school-based treatment component, as school-based treatment may be out of reach in psychiatric settings.

The present meta-analysis aims to fill the described knowledge gaps. We aimed to evaluate the treatment effects of PMT compared to waiting list (WL) or TAU for children with a mean age of 3 to 17 years, with clinical levels of disruptive behaviors in studies with a randomized controlled design. We evaluated differences in treatment effectiveness between PMT and PCIT, and between PMT and PMT with child CBT. Outcome measures examined were parent-, teacher- and clinician-rated disruptive behavior, social skills, parenting skills, parental sense of competence, and parental stress. Treatment time, treatment sessions, gender, age, and study quality as moderators of treatment effects were also examined. The following research questions were formulated:

1. How effective is standard PMT and PMT with the child involved in the treatment (i.e., PCIT, PMT with child CBT) in treating children with clinical levels of disruptive behavior at post-treatment and follow-up?
2. Is there a difference in effectiveness between standard PMT and PMT with the child involved in the treatment (i.e., PCIT, PMT with child CBT)?

## Methods

### Eligibility Criteria (PICOS)

#### Participants

Inclusion criteria were studies with children with a mean age between 3 and 17 years. The children had to have disruptive behavior problems at a clinical level, either defined as fulfilling criteria for a diagnosis of ODD or CD, or disruptive behavior problems over clinical cut-off on a well-known and established teacher or parent rating scale of disruptive behavior. Two studies were included where 1.5 SD below the mean was well above the clinical cut-off [33, 34]. Preventive studies targeting universal or selective populations with non-clinical, subclinical, or borderline behavioral problems were excluded. Studies including comorbid diagnoses such as ADHD were accepted as long as the children also had ODD, CD, or behavior problems at a clinical level, as defined above. Studies in which the participating children were developmentally or cognitively delayed, or suffered from disorders other than ODD or CD, were excluded. Studies where children were referred for maltreatment or were living with foster parents were excluded.

#### Interventions

The interventions evaluated were: (1) Standard PMT (in this meta-analysis defined as PMT directed towards parents and including core PMT treatment components [8]; (2) Parent–Child Interaction Therapy (PCIT; full model or abbreviated); (3) PMT combined with child CBT (PMT with child CBT). Treatments had to consist of at least 3 h of therapist-client contact. Studies evaluating the effects of medication were excluded.

#### Comparisons

PMT, PCIT, and PMT with child CBT were individually compared to WL. PMT was also compared directly within the same study to PMT with child CBT. In addition, the effects of PMT versus WL, PCIT versus WL, and PMT with child CBT versus WL were compared in moderator analyses.

#### Outcome Measures

Primary outcomes were measures of behavioral problems rated by parents, teachers, children, and clinicians post-treatment and at follow-up 6 or more months post-treatment. We included instruments with adequate psychometric properties measuring disruptive behavior problems. The following measures of disruptive behavior were included in the dataset: Child Behavior Checklist and Teacher Rating Form (CBCL; Externalizing, Aggression and Delinquent subscales) [35]; Eyberg Child Behavior Inventory (Intensity scale) [36]; Parent Daily Report (PDR) [37]; Strengths and Difficulties Questionnaire (SDQ CD scale) [38]; Disruptive Behavior Rating Scale (DBD; ODD subscale) [39]; Behavior Assessment System for Children 2 (BASC-2) [40]; Preschool and kindergarten behavior scales (Externalizing scale) [41]; Behar Preschool Behavior Questionnaire (PBQ) [42]. Measures of behavior problems in combination with other conditions, such as ADHD or anxiety, were not included.

Secondary outcomes were measures of social skills. The following measures were included: The Social Competence Scale (PCOMP) [43]; Social Skills Rating Scale (SSRS) [44]; Strengths and Difficulties Questionnaire (SDQ Prosocial subscale) [38]; Child Behavior Checklist and Teacher Rating Form (CBCL Social competence subscale, Teacher Rating Form Prosocial) [35]; Social Competence and Behavior Evaluation (SCBE) [45]; Parent Daily Report (Prosocial scale) [37].

We also included measures of parental strategies: the Parenting Practices Interview (PPI) [46], the Alabama Parenting Questionnaire [47], and the Arnold Parenting Scale [48]. Furthermore, we included measures of parental stress, the Parenting Stress Index [49], and a measure of the parent's sense of competence, the Parents Sense of Competence scale (PSOC) [50].

Apart from rating scales, we also included three measures of clinician-rated observation of parent–child interaction: Revised Family Observation Schedule (FOS-RIII) [51]; Gardner’s Procedure for Home Observation [52]; Dyadic Parent–Child Interaction Coding System (DPICS) [53].

#### Study Design

Randomized controlled trials with randomization at the individual or site level were included. Studies had to be published in English-language peer-reviewed journals.

### Literature Search

Database searches were conducted on four occasions: December 2014, April 2016, October 2017, and April 2019, and aimed to include all published studies. The databases used were Medline (Ovid), Psychinfo (Ovid), ERIC/ProQuest (Ovid), Cochrane (Wiley), PubMed (Complementary search), Web of Science (Thomson Reuters), Scopus (Elsevier), Cinahl (Ebsco), SweMedcombined, and Embase (Embase). Search strategies for the different databases are presented in Supplementary file 1. We also hand-searched papers that were referred to in other papers or cited in earlier meta-analyses.

### Study Selection

In total, 5106 articles were identified. A total of 4491 articles were excluded at the abstract level and 578 after full-text reading, which left 37 eligible articles. Nine of these articles, involving comparisons with TAU, were subsequently excluded since too few RCTs per comparison were identified. Ultimately, 25 RCTs were included [27–29, 31, 33, 34, 54–73], with two of them [28, 71] having complementary outcome data in three additional articles [74–76], bringing the total number of articles to 28.

**Fig. 1:**
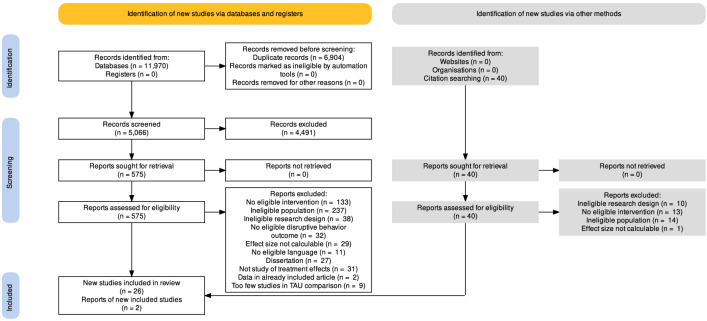
Flowchart of study selection. In the end, 28 articles were included, in total describing data from 25 different RCTs. The flowchart was created using an online tool for generating PRISMA flowcharts [77].

All titles and abstracts were screened by the first and last author (MH and PE). Studies were selected for reading in full-text if the inclusion criteria were fulfilled: age over 3 (mean) and below 18 years, PMT, RCT, clinical level of disruptive behavior. Studies selected at this phase were first reviewed in a full-text format by the first author (MH) to confirm that the inclusion criteria were fulfilled. All included studies were subsequently controlled by the authors, PE, DW, LGÖ, and by two research assistants. All articles that were excluded during the full-text reading stage were discussed by the first and last author. Causes for exclusion were documented for each study. An overview of the inclusion process and reasons for exclusion can be seen in the flow chart, Fig. 1.

### Data Extraction

Effect data (i.e., information about means, standard deviations, and numbers of treated individuals) were extracted by the first author and research assistants and subsequently reviewed by DW and PE. In two cases where articles did not provide data that could be extracted and the article was less than 11 years old, authors were contacted and asked to share information about means, standard deviations, and numbers treated. The contacted authors shared their data [28, 58]. In some studies, follow-up results and different outcomes for a study were published separately. When relevant, these results were extracted and added to the original study.

### Data Items

For the primary analyses, the following information was extracted from each included study: intervention type, comparison, measurement name, type of informant (parent, teacher, child, or clinician), and effect data. In order to evaluate effects of child and study characteristics, we extracted characteristics of the participants (mean age in years; % boys), intervention characteristics (treatment format; number of sessions; total treatment time, i.e., total number of treatment hours; treatment duration in weeks), and study origin.

### Summary Measures

Summary measures at post- and follow-up were between-group effect sizes between conditions in the same studies. Hedges’ *g* was calculated using the R package compute.es (version 0.2.4) [78] taking the mean difference between the treatments, dividing it by the pooled standard deviation, and multiplying the result with a correction factor designed to counteract upward bias in small samples. In the case multiple measures and time points were reported in the study, all data were classified into the outcome categories of interest, generating multiple effect sizes per study.

### Synthesis of Results

The majority of studies in the present meta-analysis included multiple measures, and in some cases also multiple treatment arms. The within-study correlation was handled using robust variance estimation (RVE) [79], which is considered standard best practice for meta-analyses [80]. This technique can handle dependent data and, thus, permits us to include multiple effect sizes and multiple comparisons from the same study sample without breaking any assumptions of the model. In analyses employing RVE, multiple effect sizes are reweighted using an approximate variance–covariance matrix, resulting in valid point estimates and significance tests even when the variance–covariance matrix of effect sizes within studies remains unknown. All analyses were estimated assuming an inter-correlation within studies of q = 0.8 and a random-effects model was used. Analysis was made in RStudio using the package robumeta with small sample adjustment (version 2.0) [81]. According to Tipton [82], the RVE estimators perform best when the Satterthwaite degrees of freedom are greater than 4. When the degrees of freedom were below 4 (in figures marked with ^a^), a value of p < 0.01 was used instead of p < 0.05 to avoid type I error, as suggested in Tanner-Smith et al. [83]. We chose to present the results when at least three studies were included in an analysis.

We conducted separate meta-analyses for the different types of outcomes judged to represent different underlying constructs: disruptive behavior, social skills, parenting skills, parental sense of competence (data found only in the standard PMT versus WL comparison), and parental stress. Parenting skills were divided into positive parenting skills (use of positive skills such as praise and rewards), and negative parenting skills (use of negative strategies such as harsh, overreactive, or submissive parenting) in order to detect differences in treatment effect between these two constructs. Furthermore, the effects of PMT versus WL, PCIT versus WL, and PMT with child CBT versus WL were compared in moderator analyses, also using robust variance estimation. For these moderator analyses, an alternative method is network meta-analysis in which effect sizes from all arms of a study can be incorporated rather than just from a single comparison. This would be relevant for the two studies [28, 29] in our dataset that used three-arm designs. We replicated our moderator analyses using this method with one outcome measure for each study, finding a similar pattern of results (contact authors for further details).

Moderator analyses, determining potential effects of child and study characteristics, were conducted by means of a meta-regression, but only for the standard PMT versus WL comparison at post-treatment on the disruptive behavior outcome, as the heterogeneity was judged large enough with an *I*^2^ between 50 and 70% [84]. All moderators (i.e., mean age in years, % boys, total treatment time in hours, study quality) were analyzed in the same model following current best practice [80]. In a subsequent analysis, total treatment time in hours was substituted with the number of treatment sessions.

### Assessment of Study Quality and Risk of Bias

The psychotherapy outcome study methodology rating scale [85] and the Cochrane risk-of-bias tool [86] were used for assessment of methodological study quality.

The psychotherapy outcome study methodology rating scale consists of 22 items: (1) Clarity of sample description, (2) Severity/chronicity of the disorder, (3) Representativeness of the sample, (4) Reliability of the diagnosis in question, (5) Specificity of outcome measures, (6) Reliability and validity of outcome measures, (7) Use of blind evaluators, (8) Assessor training, (9) Assignment to treatment, (10) Design, (11) Power analysis, (12) Assessment points, (13) Manualized, replicable, specific treatment programs, (14) Number of therapists, (15) Therapist training/experience, (16) Checks for treatment adherence, (17) Checks for therapist competence, (18) Control of concomitant treatments, (19) Handling of attrition, (20) Statistical analyses and presentation of results, (21) Clinical significance, (22) Equality of therapy hours (for non-WL designs only). The scale generates a summary score per study. Each item is rated as 0 (poor), 1 (fair), or 2 (good), allowing for a range of 0–44 points. In the present meta-analysis mean study quality score was 21.6 (SD 4.75) with an overall range of 13–33. Scores for each study can be seen in Table 1. The ratings of study quality were made by trained research assistants with no connection to the evaluated studies. The inter-rater reliability, based on 20% randomly selected studies, was ICC = 0.88 for the total score, indicating good inter-rater reliability. Differences between raters were discussed in order to reach agreement.

**Table 1:** Descriptives of the studies and treatment conditions included in the meta-analysis

Author	Country	Treatment	Comparison	Assessment	Rater	Treatment time PMT	Quality score	N	% Boys	Age range	Mean age	Therapy format	PMT method	PMT manual
Axberg, 2012	Sweden	PMT	WL	Post	P, T	24	25	62	84	4–8	6.0	G	IY	Webster-Stratton, 2001
Barkley, 2000	USA	PMT	WL	Post	P	30	19	81	64	4–6	4.8	G	Barkley	Barkley, 1997
Braet, 2009	Belgium	PMT	WL	Post	P, T	22	13	64	64	4–8	5.6	G	PMT	-
Brestan, 1997	USA	PMT	WL	Post	P	-	16	30	83	3–6	4.5	I	PCIT	Eyberg & Boggs, 1989
Carvalho Homem, 2015	Portugal	PMT	WL	Post	P, Clin	28	23	83	73	3–6	4.5	G	IY	Webster-Stratton, 2001
David, 2014	Romania	PMT	WL	Post	P, T	15	24	85	48	4–12	6.0	G	PMT	Clark, 1996
Enebrink, 2012	Sweden	PMT	WL	Post	P	11	23	104	58	3–12	6.8	Int	KOMET	Kling, 2006
Eyberg, 1995	USA	PMT	WL	Post	P, Clin	13	17	22	80	3–6	4.5	I	PCIT	Eyberg & Durning, 1994
Frank, 2015	New Zealand	PMT	WL	Post, 6 mon	P	11.5	18	42	69	3–8	5.6	G	Triple P	Sanders, 2012
Gardner, 2006	UK	PMT	WL	Post	P, Clin	28	20	76	74	2–9	5.9	G	IY	Webster-Stratton, 2001
Helander, 2018	Sweden	PMT	PMT + child- CBT	Post	P	28	27	120	73	8–12	9.3	G	KOMET	Kling, 2006
Hutchings, 2007	UK	PMT	WL	Post	P, Clin	30	15	153	60	3–5	3.8	G	IY	Webster-Stratton, 2001
Kazdin, 1992	USA	PMT	PMT + child- CBT	Post, 12 mon	P, Child	28	25	68	78	7–13	10.3	I	PMT	Kazdin, 1987
Larsson, 2009^1^	Norway	PMT, PMT + child- CBT	WL, PMT + child- CBT	Post, 12 mon	P, T, Child	24	30	127	80	4–8	6.6	G	IY	Webster-Stratton, 2001
Leung, 2015	Hong Kong	PMT	WL	Post	P, Clin	-	24	111	74	2–7	4.5	I	PCIT	Eyberg & Funderburk, 2010
Markie-Dadds, 2006	Australia	PMT	WL	Post	P	3	23	26	76	2–6	3.9	I	Triple P	Sanders, 1999
McGilloway, 2012	Ireland	PMT	WL	Post	P, Clin	28	21	149	62	2–7	4.8	G	IY	Webster-Stratton, 2001
Nixon, 2003	Australia	PMT	WL	Post	P, Clin	22	22	54	70	3–6	3.9	I	PCIT	Hembree-Kigin & McNeil, 1995
Pepler, 2010	Canada	PMT + child- CBT	WL	Post	P, T	18	20	87	0	5–11	8.6	G	SNAP	Augimeri, 2007
Sanders, 2000	Australia	PMT	WL	Post	P, Clin	10	24	136	68	3–4	3.4	G	Triple P	Sanders, 1999
Schuhman,1998	USA	PMT	WL	Post	P, Clin	13	17	64	81	3–6	4.9	I	PCIT	Eyberg & Durning, 1994
Scott, 2001^1^	UK	PMT	WL	Post, 48 mon	P	30	19	141	74	3–8	5.7	G	IY	Webster-Stratton, 2001
Webster-Stratton, 2004	USA	PMT	WL	Post	P	42	33	57	90	4–8	5.9	G	IY	Webster-Stratton, 2001
Webster-Stratton, 1997	USA	PMT, PMT + child- CBT	WL, PMT + child- CBT	Post, 12 mon	P, T, Child Clin	36	25	70	74	4–7	5.7	G	IY	Webster-Stratton, 2001
Zangwill, 1983	USA	PMT	WL	Post	P, Clin	14	16	11	-	2–8	3.0^2^	I	PCIT	Hanf & Kling, 1974

Treatment = The active treatment; Comparison = The condition that the active treatment is compared against; PMT = Parent Management Training; PMT + child-CBT = PMT with child CBT; WL = Waiting list; Assessment = Time point of between group effect size difference at post assessment and number of months post treatment assessment; Rater: P = Parent T = Teacher, Child = Child, Clin. = Clinician rated outcomes/observations; Treatment time PMT = Approximate number of hours with treatment; Quality score = Rating of quality according to the psychotherapy outcome study methodology rating scale (Öst 2008); N = Number of children participating in the relevant conditions in each study; Therapy format: G = group, I = Individually, Int. = Internet with therapist contact at least 3 h; PMT Method: IY = Incredible Years, PCIT = Parent Child Interaction Therapy, SNAP = Stop Now And Plan. ^1^For two of the studies [28, 71], complimentary data was found [74–76], not presented in this table. ^2^ Median age

In line with the Cochrane risk of bias tool (RoB) [86], the studies were coded “low”, “some”, and “high” risk in respective domains, and a summary risk of bias was estimated. In total, 6 studies were coded as having high, 19 some, and zero had low risk. For the domain randomization process, all studies were randomized controlled studies but did not report how allocation sequence was generated or whether allocation was concealed (13 studies were coded as low risk, 10 some risk, and 2 high risk). For the domain Deviation from intended intervention, most of the studies reported no deviation (17 low, 5 some, and 3 high risk). As for the domain missing outcome data, bias was detected in half of the studies (12 low, 9 some, and 4 high risk). Regarding the domain Measurement of the outcome, as in many studies on the effects of PMT, the parents were aware of the treatment they received and were the main informants of program effects (25 some risk). For the domain Bias in selection of the reported results, the majority of studies were conducted before registration of study protocol became mandatory (5 low and 20 some risk). Interrater reliability was assessed in four out of the 25 articles. The total proportion of agreement was 0.85, with 17 out of 20 items agreed upon. Individual variables had the following proportions of agreement: Randomization = 1, Deviation from intended treatment = 0.75, Missing outcome data = 0.75, Blinding outcome measurement = 1, Selection of reported results = 0.75.

Publication bias, the tendency to report and publish only large and significant effects constitutes a risk to external validity in a meta-analysis. Common methods used to analyze possible publication bias are funnel plots and Egger’s test of funnel plot symmetry. However, as these methods have been shown to perform less well in meta-analysis with multiple and dependent effect sizes [80, 87] they were not performed.

### Sample Characteristics

A total of 2023 individuals participated in the included studies. The mean age was 5.5 years and the age range was 2–13 years (no studies with children above 13 years of age were found). Sixty-nine percent were boys (see Table 1). In seven out of 25 studies, the proportion of children with comorbid ADHD was presented ranging from 3 to 82% (mean 55%). The 25 studies found were published between 1983 and 2018 and were conducted in 12 countries, representing four continents. In 16 studies, standard PMT was compared to WL. Six studies compared PCIT to WL. In three studies, PMT with child CBT was compared to WL. Four studies compared PMT to PMT with child CBT. A few studies included multiple comparison groups (see Table 1). Additional information on baseline levels of disruptive behavior, separately for PMT, PCIT, and PMT with child CBT can be found in Supplementary file 2, Table S1.

Unfortunately, only two studies were found with follow-up assessments in the PMT versus WL comparison [61, 88]. The only comparison condition where three or more studies included follow-up assessments was the PMT versus PMT with child CBT comparison (*n* = 3).

Parent-rated outcomes were found in all studies. In one comparison, PMT with child CBT versus WL, we were able to analyze teacher-rated outcomes. Child-rated outcomes were too few to analyze. Clinician-rated outcomes were found in the standard PMT versus WL, PCIT versus WL, and PMT with child CBT versus WL comparisons, but not in the PMT versus PMT with child CBT comparison.

## Results

### How Effective is Standard PMT and PMT with the Child Involved in the Treatment?

**Fig. 2:**
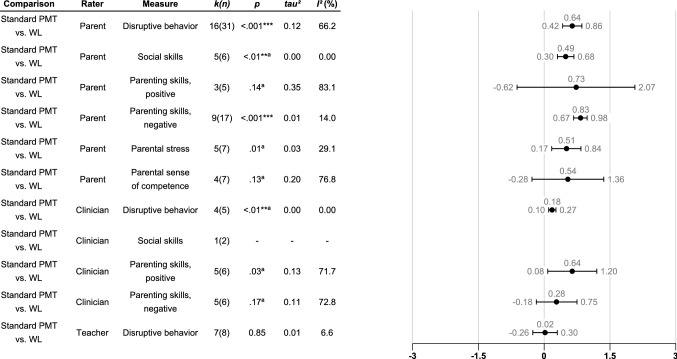
Robust variance estimations of between group effect sizes (Hedges’ g) of standard PMT compared to WL at post-treatment assessment for different raters and measures. Only results from analyses comprising more than two studies are shown. Error bars denote the 95% confidence intervals. Comparison = The comparison that is being investigated; Rater = The type of rater that has contributed with the dependent measure; Measure = The dependent measure that is being investigated; *k(n) *= Number of studies/number of effect sizes; *p* = The *p*-value. *p*-values marked with “a” means that they are unstable due to degrees of freedom being below 4. In these cases, only *p*-values below .01 are regarded as significant. *p <.05, **p <.01, ***p <.001; *tau*^2^ = Between study variance; *I*^2^ (%) = Percentage of variation across studies that is due to heterogeneity rather than to chance.

As can be seen in Fig. 2, standard PMT was significantly more effective than WL, with medium effect sizes on parent-rated measures of child disruptive behavior (k = 16) and social skills (k = 5), and a large effect size on negative parenting skills (k = 9). For positive parenting skills (k = 3), parental stress (k = 5), and parental sense of competence (k = 4), standard PMT was not significantly more effective than WL, although the effect sizes were in the expected direction. Forest plots can be found in Figs. S1-S10 Supplementary file 2.

**Fig. 3:**
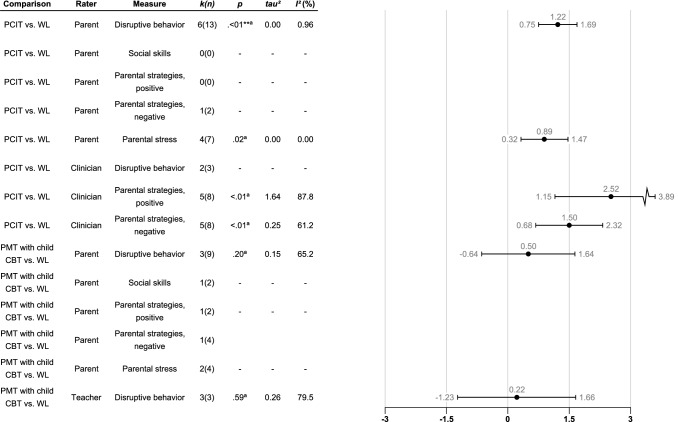
Robust variance estimations of between group effect sizes (Hedges’ g) of PMT with the child included in the treatment (PCIT or PMT with child CBT) compared to WL at post-treatment assessment for different raters and measures. Only results from analyses comprising more than two studies are shown. Error bars denote the 95% confidence intervals. Comparison = The comparison that is being investigated; Rater = The type of rater that has contributed with the dependent measure; Measure = The dependent measure that is being investigated; *k(n)* = Number of studies/number of effect sizes; *p* = The *p*-value. *p*-values marked with “a” means that they are unstable due to degrees of freedom being below 4. In these cases, only *p*-values below .01 are regarded as significant. *p <.05, **p <.01, ***p <.001; *tau*^2^ = Between study variance; *I*^2^ (%) = Percentage of variation across studies that is due to heterogeneity rather than to chance.

In teacher-rated disruptive behavior outcomes, examined in seven studies, no significant effect was found regarding disruptive behavior. We found a significant effect size for clinician-rated disruptive behavior, favoring PMT when compared to WL (evaluated in four studies). For clinician-rated parenting skills, no significant differences were found in this small sample.

Too few studies were found to analyze follow-ups of six months or longer. In standard PMT compared to WL, only two studies included follow-up data. Therefore no analysis was conducted of longer-term effects.

When examining parent-rated effectiveness of PCIT (six studies), PCIT was significantly more effective compared to WL with large effect sizes for reduced disruptive behavior and parental stress (see Fig. 3; forest plots can be found in Figs. S11-S16 Supplementary file 2). Regarding clinician-rated parent–child interactions, examined in five studies, the effect size of positive and negative parental strategies were large and significant for PCIT compared to WL. We also examined three studies where PMT combined with child CBT was compared to WL. No significant effects were found in parent- or teacher-rated outcomes. Too few studies were found to analyze follow-ups of six months or longer in PCIT or PMT with child CBT versus WL.

### Is There a Difference in Effectiveness Between Standard PMT and PMT with the Child Involved in the Treatment?

**Fig. 4:**
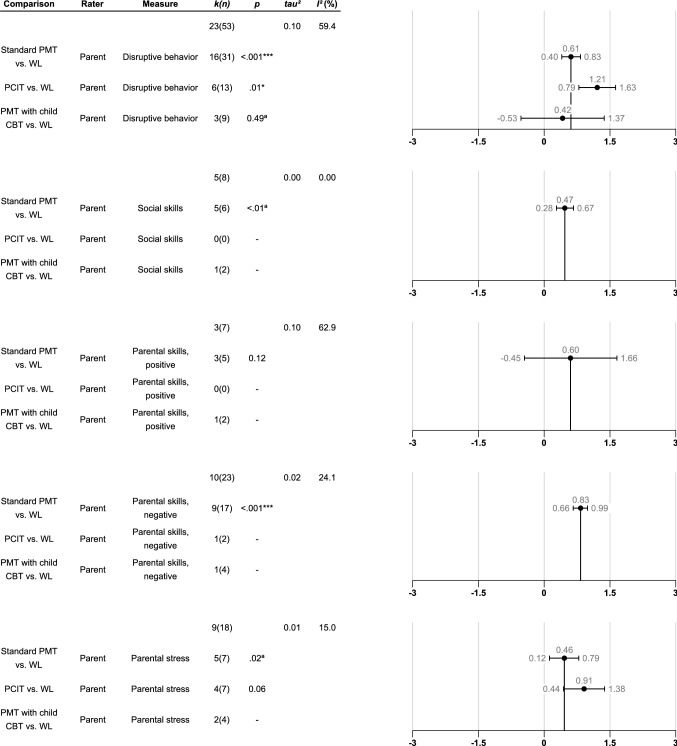
Robust variance estimation moderator analyses of type of comparison for between group effect sizes (Hedges’ g) at post-treatment parent assessment for different measures. In total, five different analyses are presented. The first row of each analysis gives information about overall *k*, *n*, *τ*^2^, and *I*^2^, whereas the second row is the intercept and the subsequent rows denote and test for the difference from that intercept. In the presentation of these analyses, the intercept effect sizes have been added to the subsequent effect sizes and confidence intervals in order to simplify the interpretation. Only comparisons that include more than two studies are shown. Error bars denote the 95% confidence intervals. Comparison = The comparison that is being investigated; Rater = The type of rater that has contributed with the dependent measure; Measure = The dependent measure that is being investigated; *k(n)* = Number of studies/number of effect sizes; *p* = The *p*-value. *p*-values marked with “a” means that they are unstable due to degrees of freedom being below 4. In these cases, only *p*-values below .01 are regarded as significant. *p <.05, **p <.01, ***p <.001; *tau*^2^ = Between study variance; *I*^2^ (%) = Percentage of variation across studies that is due to heterogeneity rather than to chance.

**Fig. 5:**
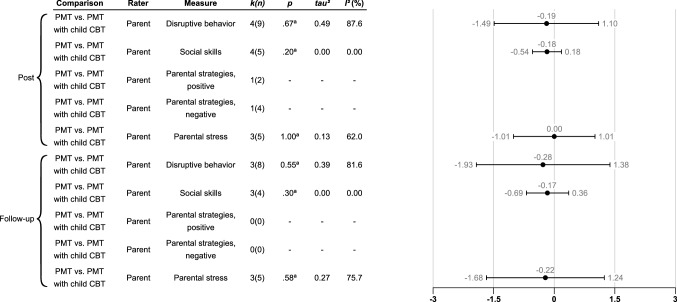
Robust variance estimations of between group effect sizes (Hedges’ g) of parent rated standard PMT compared to PMT with child CBT for different measures at different timepoints. Only results from analyses comprising more than two studies are shown. The first five rows denote post-treatment scores while the last five rows denote follow-up scores (12 months after treatment completion). Error bars denote the 95% confidence intervals. Comparison = The comparison that is being investigated; Rater = The type of rater that has contributed with the dependent measure; Measure = The dependent measure that is being investigated; *k(n)* = Number of studies/number of effect sizes; *p* = The *p*-value. *p*-values marked with “a” means that they are unstable due to degrees of freedom being below 4. In these cases, only *p*-values below .01 are regarded as significant. *p <.05, **p <.01, ***p <.001; *tau*^2^ = Between study variance; *I*^2^ (%) = Percentage of variation across studies that is due to heterogeneity rather than to chance.

We were also interested in examining if there was increased effectiveness of PMT when the child was included in the treatment, as in PCIT and PMT with child CBT. The results are presented in Figs. 4 and 5. First, we ran a moderator analysis with the type of PMT as a moderator (standard PMT, PCIT, and PMT with child CBT) and analyzed treatment effects of the three versions of PMT compared to WL (see Fig. 4). Results showed that the effect of PCIT versus WL was significantly larger compared to standard PMT versus WL in reducing disruptive behavior, while the effect of PMT with child CBT versus WL did not differ significantly from the effect of standard PMT versus WL. In parental stress outcomes, PCIT versus WL showed a non-significant larger effect compared to standard PMT versus WL. One possible explanation of the differences between PCIT and standard PMT could be related to the age of the children. In our analysis, the mean age in the PCIT studies (4.22, SD 0.68) and the standard PMT studies (5.30, SD 1.00) were significantly different. Another difference between PCIT and standard PMT is that the treatment time may differ. However, there was no significant difference in the number of treatment sessions between PCIT (M = 13.25, SD = 1.57) and PMT (M = 12.75, SD = 4.06).

We were also able to examine the effects of standard PMT compared directly to PMT with child CBT at post-measurement within four studies (see Fig. 5; forest plots can be found in Figs. S17-S22 Supplementary file 2). No significant differences in effect sizes were discovered in disruptive behavior outcomes and there were large variations in effect sizes among the studies in all outcomes.

In the comparison between standard PMT versus PMT with child CBT, three studies had a follow-up assessment. At one-year follow-up effect sizes were small and non-significant, with large variation among studies.

### Moderator Analyses

In order to determine whether child characteristics, treatment characteristics, or study quality moderated treatment results, a meta-regression analysis of the effect size for standard PMT compared to WL was used (see Table 2). No significant effects were found for the variables age, sex, and treatment time in hours, indicating that these factors did not moderate treatment effectiveness. Since group- and individual treatment may differ in the amount of time that is directed to a specific family, we also analyzed whether the number of treatment sessions (instead of treatment time in hours) moderated the effect, which was not the case (not reported). Study quality, determined by the psychotherapy outcome study methodology rating scale [85] was found to moderate treatment effect significantly, with higher study quality being associated with a larger effect size.

**Table 2:** Moderator analysis of age, gender, treatment time, and study quality on parent's ratings of disruptive behavior for PMT vs. WL

Measure		*k(n)*	Beta	95% CI	*p*	*tau*^*2*^	*I* (%)
		16(31)				0.08	55.6
Disruptive behaviour	Intercept		0.74	0.51, 0.98	< .001***		
	Mean age		− 0.06	− 0.33, 0.21	.56		
	Proportion boys		0.06	− 0.19, 0.30	.54^a^		
	Treatment time		− 0.20	− 0.55, 0.15	.20		
	Study quality		0.27	0.08, 0.46	.01*		

*k(n)* = Number of studies/number of effect sizes; Beta = indicates the value of the slope for each continuous variable; ^a^ = *p*-value is unstable due to degrees of freedom below 4, therefore, a *p*-value at .01 is regarded as non-significant whereas a *p*-value below .01 is regarded as significant; *p < .05, **p < .01, ***p < .001; *tau*^*2*^ = between study variance; *I*^2^ (%) = percentage of variation across studies that is due to heterogeneity rather than to chance

## Discussion

This meta-analysis exclusively investigated the effectiveness of PMT on clinical levels of disruptive behavior without inclusion of other treatments, synthesizing findings from 25 RCTs. Our first research question focused on the effectiveness of standard PMT. We can conclude that standard PMT targeting children with clinical levels of disruptive behavior was significantly more effective at post-treatment with a medium effect size compared to WL. The effect size found in this study of standard PMT compared to WL was somewhat larger compared to meta-analyses that also included subclinical levels of disruptive behavior [11, 12, but see also 9]. Our effect size was closer to the ones obtained by Furlong et al. [13] and Fossum et al. [89] showing medium effect sizes on clinical levels of disruptive behavior for PMT compared to WL. This may suggest a larger effect of standard PMT when treating clinical levels of disruptive behavior as compared to non-clinical levels of disruptive behavior.

Only a few standard PMT studies included follow-up data in both treatment and comparison conditions, prohibiting comprehensive analyses. Although a previous meta-analysis using within-group effects has shown sustained effects of PMT over time [12], it is clear that more RCTs on PMT effectiveness on clinical levels of disruptive behavior with follow-up data in both comparisons are needed before firm conclusions can be drawn.

This meta-analysis initially had the ambition to also investigate PMT versus TAU. Surprisingly, only two RCTs were found per comparison (standard PMT versus TAU, PCIT versus TAU, and PMT with child CBT versus TAU), highlighting an important knowledge gap in the literature and stressing that more studies are called for. A low number of relevant studies was also evident when attempting to analyze PMT with child CBT compared to WL, resulting in unreliable effects, thereby prohibiting conclusions to be drawn.

We were interested in evaluating the effects of PMT when the child was involved in treatment. We found a large and significant post-treatment effect size for disruptive behavior for PCIT compared to WL. These results are in line with a previous meta-analysis on PCIT [23]. The present meta-analysis contributes by extending the results to clinical levels of disruptive behavior. In contrast, PMT combined with child CBT was not significantly more effective than WL. The lack of reliable effect, albeit a medium effect size in the expected direction, may be related to that the number of studies were few.

Our findings on disruptive behavior confirm the results of previous studies investigating the effect of PMT versus WL. Moreover, we found a reliable effect on social skills for PMT. This is notable as it shows that parents perceive their child’s social ability to have improved following treatment, in spite of the child not being active in the treatment or social skills being specifically targeted. Similar findings have been reported by Battagliese and colleagues [14]. More expected and also found in previous meta-analyses [13, 15] were that negative parenting skills (PMT) and parental stress (PCIT) improved.

Our second research question examined if there was a difference between standard PMT and PMT with the child involved in the treatment. Our results showed significantly larger effect sizes for PCIT versus WL compared to PMT versus WL, suggesting that PCIT could be more effective than standard PMT in the treatment of clinical levels of disruptive behavior. PCIT is generally used in the treatment of younger children, 2–7 years old, while standard PMT is designed for children between 3 and 12 years old. In accordance with this, our analysis showed that the mean age of children in PCIT studies was lower than the mean age in PMT studies. Thus, it cannot be ruled out that the difference in treatment effects is related to the age difference. Although the treatment time may differ between PCIT and standard PMT, this was not the case here, suggesting that the number of treatment sessions does not explain the larger effect for PCIT. The larger treatment effect might also be explained by the individual delivery format in PCIT, which enables individual tailoring to the family. In a previous meta-analysis, individually delivered PMT has been found to be superior to group-delivered PMT [90]. In the present meta-analysis, the individually delivered PMTs were to a large extent PCIT studies, which prohibited us from systematically investigating the importance of an individual format among standard PMT. Our results are supported by a meta-analysis [91], showing that PCIT tended to have larger effect sizes on parent-rated disruptive behavior compared to one of the standard PMT programs (Triple-P) on clinical and subclinical child disruptive behavior. Nonetheless, more studies with direct comparisons of PMT and PCIT are needed before firm conclusions on the effectiveness of PCIT compared to PMT can be drawn.

Contrary to our expectations, PMT with child CBT compared to WL did not significantly differ from standard PMT compared to WL at post-treatment. Furthermore, when PMT was compared directly to PMT with child CBT, no significant effects were found at post-treatment or one-year follow-up on parent-rated outcomes. There was large variability in effect sizes and few studies comparing PMT with child CBT to standard PMT, suggesting that more studies are needed in order to bring clarity to the potential additive, or lack of additive, effects of child CBT to PMT.

Teacher ratings of disruptive behavior were provided in a limited number of studies, showing no significant effects in any comparison. The variability among studies was large and *p*-values were unstable. Previous studies indicate that there is typically low correspondence between teacher- and parental ratings of disruptive behavior [92], one potential reason being that disruptive behavior can be more prominent at home compared to school. Symptoms can be present in only one setting and still constitute major distress with such a low level of functioning that the disruptive behavior is considered to be at a clinical level. In the RCTs included in this meta-analysis, teachers were not involved in the treatments. Our results suggest that the effect does not automatically generalize to the school when the school is not involved in the treatment.

In a total of eleven studies (six PMT and five PCIT), clinicians had observed parent–child interactions. The results in the clinician-rated outcomes were largely in concordance with parent-rated measures on child behavior, which supports the validity of the parent-rated outcomes for this outcome. For PMT, parent- and clinician-rated positive and negative parenting skills were in the same direction, although not always with significant effects. The association between parent-reported and observed parenting behavior has recently been examined in a multilevel meta-analysis indicating a weak but significant overall correlation [93]. When it comes to the association between parent- and observer ratings on child disruptive behavior, a study showed high discrepancy between parent-rated and clinician-rated disruptive child behavior with parents scoring higher levels of disruptive behavior than observers [94]. In this study children with sub-clinical levels of disruptive behavior were included, which could, hypothetically, help explain the discrepancy with our results.

Previous meta-analyses on the efficacy of PMT that included non-clinical levels of disruptive behavior and/or various forms of treatment designs did not find a moderator effect of age [20, 95] or gender [20, 89] on PMT effectiveness. Our study confirms these findings in clinical samples. Treatment time in hours has not been explored specifically in earlier meta-analyses, however, analyses of number of treatment sessions have indicated no moderating effect [20, 90], which corresponds well to our finding that neither treatment times in hours nor number of sessions moderated standard PMT treatment effectiveness.

We found that higher study quality was associated with a higher effect size on standard PMT compared to WL. In contrast, a meta-analysis by McCart [18] including non-randomized studies and mostly clinical levels of disruptive behavior, found that improved study quality, as measured by a quality rating scale by Durlak et al. [96], was associated with lower effectiveness. When comparing our meta-analysis with the McCart meta-analysis [18], only seven studies (23%) of the McCart studies were included in our study, which indicates that conclusions are based on different bodies of studies, which might explain the difference in results. Furthermore, the scale used in McCart, developed by Durlak et al. [96], is not equivalent to the one we used [85]. High study quality has previously been found to be a positive moderator of CBT treatment effects in a meta-analysis on OCD treatment for children [97]. Tentatively, high-quality trials have more homogenous, representative, and well-diagnosed (e.g., structured interviews) samples, reliable and valid instruments, higher-powered studies, and specific treatment programs run by well-trained and competent therapists. It is possible that high quality on these factors may lead to less noise in the data and, therefore, to larger effects.

Risk of bias was assessed as low in approximately half of the studies concerning randomization, deviation from treatment, and missing outcome, according to the RoB tool [86], whereas the majority of studies had some risk of bias in the remaining two domains: blinding of assessors and selective reporting of data. Although all studies were randomized controlled studies, older studies did not report how allocation sequence was generated and, as always, the parents were aware of the treatment they received and were the main informants of program effects. In addition, the majority of studies were conducted before registration of study protocol became mandatory. Seeing the small number of studies with high risk, in spite of older studies being included in the meta-analysis, the results of the meta-analysis can be assumed to be valid.

### Strengths and Limitations

A major strength of this meta-analysis was the selection of RCTs that include clinical levels of disruptive behavior only, combined with a selection of studies on PMT without interference of other treatment types. Another strength of this meta-analysis is that we were able to compare standard PMT with two other versions of PMT in which the child is involved in the treatment, identifying treatment gains of bringing the child into the treatment setting. We were also able to broaden the assessment by evaluating not only disruptive behavior, but also child social skills, parental strategies, parental sense of competence, and parental stress. Finally, our results were analyzed using robust variance estimation enabling us to handle within-study and informant dependencies, thereby enhancing power and producing reliable estimations.

A limitation of the meta-analysis is that some of the planned comparisons were not possible to undertake due to the limited number of studies conducted. Even though the number of RCTs at clinical levels of disruptive behavior has increased largely, the number of studies with a TAU comparison and studies with follow-up assessments including a WL were too few to enable conclusions to be drawn. The lack of RCTs at clinical levels of disruptive behavior with a TAU comparison and with continued follow-up assessment highlights the imminent need for further studies. In addition, studies investigating the efficacy of PMT with child CBT were few, thereby limiting the conclusions that could be drawn. Furthermore, we included studies on children with disruptive behavior above a clinical cut-off based on rating scales or with a disruptive behavior disorder diagnosis, but it would have been preferable to only include studies on children with a clinician-rated diagnosis. Only seven of the 25 studies included children with a disruptive behavior disorder diagnosis, reflecting the immaturity of the field, and illustrating the need for more high-quality studies. Finally, it is possible that different baseline levels might contribute to the relative effectiveness of PCIT over PMT. We therefore compared the baseline values of the ten PMT and six PCIT studies that included Eyberg Child Behavior Inventory (ECBI) [36] measurements (i.e., six studies were not included in these analyses since they did not use the ECBI), finding no difference in baseline difficulties in behavior problems (see Table S1 Supplementary file 2 for further information).

## Conclusions

In the treatment of children with clinical levels of disruptive behavior, standard PMT is more effective than WL in reducing disruptive behavior and enhancing functional parental strategies. These findings support current treatment recommendations to offer PMT to parents of children with clinical levels of disruptive behavior. We can also conclude that PCIT, the PMT approach where the parent receives guidance and feedback from the therapist through a bug in the ear while interacting with the child, shows large effects, which should have implications for future treatment recommendations. Nonetheless, further studies comparing PCIT directly to PMT are needed.

## Summary

PMT is the recommended treatment for disruptive behavior disorder in school-aged children. Updated meta-analyses investigating the effects of PMT at clinical levels of disruptive behavior in RCTs are lacking, as are evaluations of the possible additional effects of PMT treatment with child involvement. In this meta-analysis, 25 studies and 2023 individuals were included. We synthesized RCTs of PMT compared to WL at clinical levels of disruptive behavior in children (age range 2 to 13). We also synthesized RCTs of PMT with the child involved in the treatment (i.e., PCIT and PMT combined with child CBT) compared to WL. In addition, we compared the effects of PMT combined with child CBT with PMT alone. We used random-effects meta-regression models with robust variance estimates to summarize overall effects and explore potential moderator effects. Results showed that PMT (*g* = 0.64 [95% CI 0.42, 0.86]) and PCIT (*g* = 1.22 [95% CI 0.75, 1.69]) were more effective than waiting-list (WL) in reducing parent-rated disruptive behavior, and PMT also in improving parental skills (*g* = 0.83 [95% CI 0.67, 0.98]) and child social skills (*g* = 0.49 [95% CI 0.30, 0.68]). PCIT versus WL (*g* = 1.21 [95% CI 0.79, 1.63]) had larger effects in reducing disruptive behavior than PMT versus WL (*g* = 0.61 [95% CI 0.40, 0.83]). In the few studies found, the addition of child CBT to PMT did not yield larger effects than PMT (*g* = 0.19 [95% CI − 1.10, 1.49]) or WL (*g* = 0.50 [95% CI − 0.64, 1.64]). To conclude, the present meta-analysis gives support to treatment recommendations to offer PMT to children with clinical levels of disruptive behavior and highlights the additional benefits of PCIT for younger ages.

### Supplementary Information

Below is the link to the electronic supplementary material.

Supplementary file1 (PDF 636 KB)

Supplementary file2 (DOCX 2054 KB)
