# Supplementary material for: The Efficacy of Parent Management Training With or Without Involving the Child in the Treatment Among Children with Clinical Levels of Disruptive Behavior: A Meta-analysis
Source: Child Psychiatry Hum Dev. 2022 Jul 5;55(1):164–81. doi: 10.1007/s10578-022-01367-y (PMC10796477; doi:10.1007/s10578-022-01367-y)
Supplement: Supplementary file 1 — Supplementary file1 (PDF 636 KB) [file 10578_2022_1367_MOESM1_ESM.pdf]

Table S1

Descriptive data of baseline scores for the Eyberg Child Behavior Inventory Intensity Subscale for studies, divided by type of comparison and inclusion criteria

Condition 1	Condition 2	Inclusion criteria (*k*)	Combined Mean (SD)	Condition 1 Mean (SD)	Condition 2 Mean (SD)
PMT	WL				
		ODD (2)	154 (26)	155 (24)	154 (28)
		Cut-off (8)	150 (27)	150 (27)	149 (27)
		Total (10)	151 (27)	151 (27)	150 (28)
PCIT	WL				
		ODD (3)	170 (28)	167 (25)	173 (32)
		Cut-off (3)	166 (21)	168 (23)	164 (19)
		Total (6)	168 (25)	168 (24)	167 (25)
PMT + Child CBT	WL				
		ODD (2)	153 (27)	153 (26)	154 (28)
		Cut-off (0)	-	-	-
		Total (3)	153 (27)	153 (26)	154 (28)
PMT + Child CBT	PMT				
		ODD (2)	154 (25)	153 (26)	155 (24)
		Cut-off (0)	-	-	-
		Total (2)	154 (25)	153 (26)	155 (24)

Note. Means and standard deviations are derived from sample means and sample standard deviations reported in each study using the sample.decomp function from the R package Utilities (O'Neill, 2021. utilities: Data Utility Functions. R package version 0.3.0. https://CRAN.R-project.org/package=utilities). Whenever possible, numbers are based on the scores of both mothers and fathers. However, sometimes only one of these scores are given. The Combined column is derived by combining Condition 1 and Condition 2. A robust variance estimation moderator analysis with PMT vs. WL as one level and PCIT vs. WL as the other level could not show a difference between the estimates (p=0.90). k = Number of studies; PMT = Parent Management Training; PCIT = Parent Child Interaction Therapy; PMT + child CBT = PMT with child CBT, WL = Waiting list; ODD = ODD diagnosis diagnosed by clinician using structured diagnostic assessment; Cut-off = Studies on children with disruptive behavior problems above clinical cut-off.

Figure S1

Forest plot of effect sizes making up the analysis of parents' assessment of disruptive behavior for standard PMT compared to WL at post-treatment

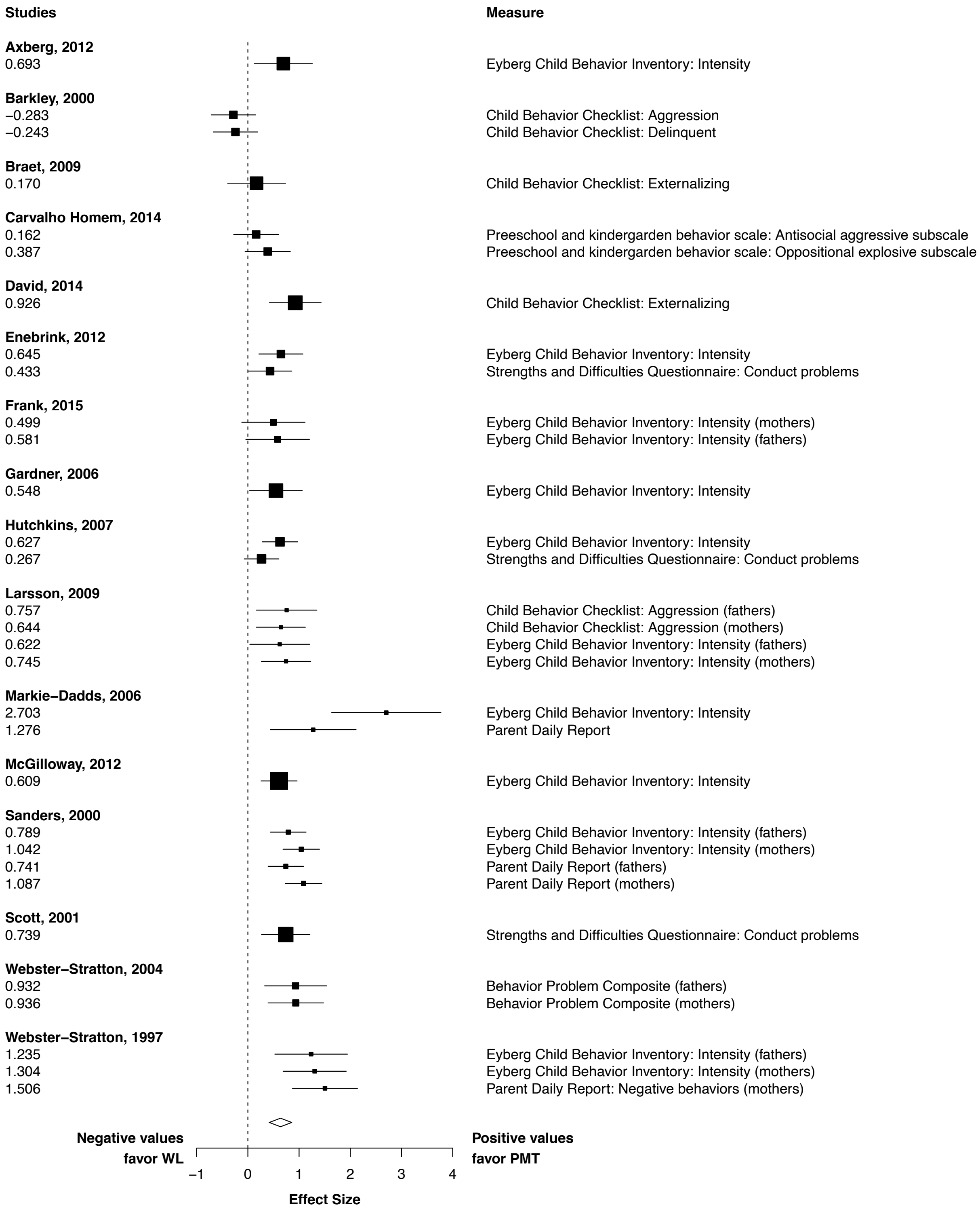

Note. The robust variance estimation of this data can be found in the first row of Figure 2.

Figure S2

Forest plot of effect sizes making up the analysis of parents' assessment of social skills for standard PMT compared to WL at post-treatment

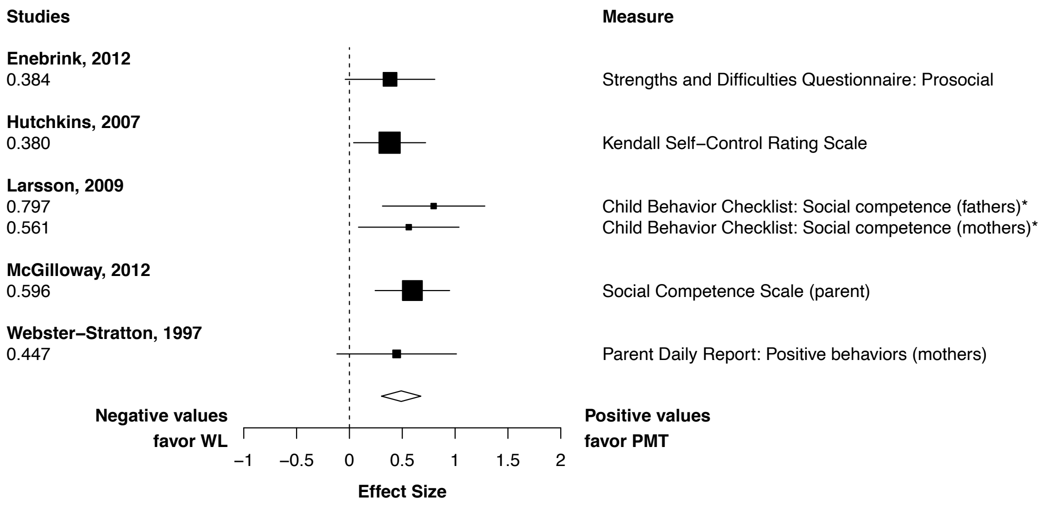

Note. The robust variance estimation of this data can be found in the second row of Figure 2. * = Data from Drugli et al., 2007.

Figure S3

Forest plot of effect sizes making up the analysis of parents' assessment of positive parenting skills for standard PMT compared to WL at post-treatment

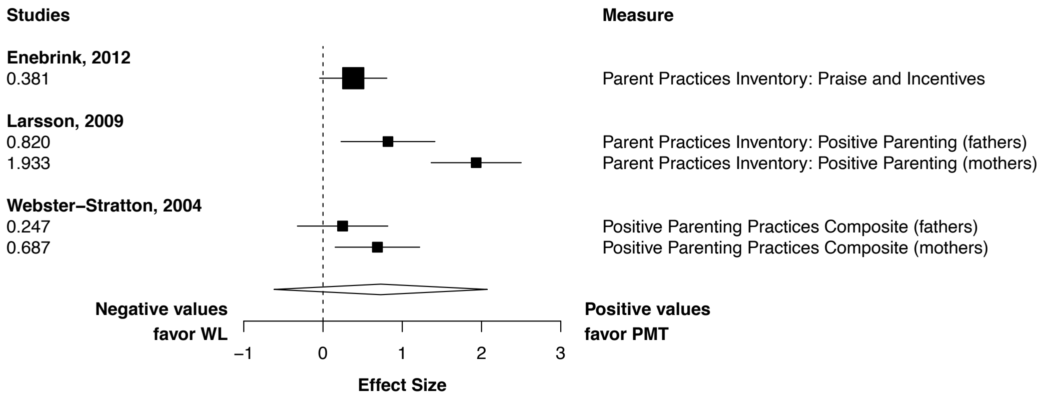

Note. The robust variance estimation of this data can be found in the third row of Figure 2.

Figure S4

Forest plot of effect sizes making up the analysis of parents' assessment of negative parenting skills for standard PMT compared to WL at post-treatment

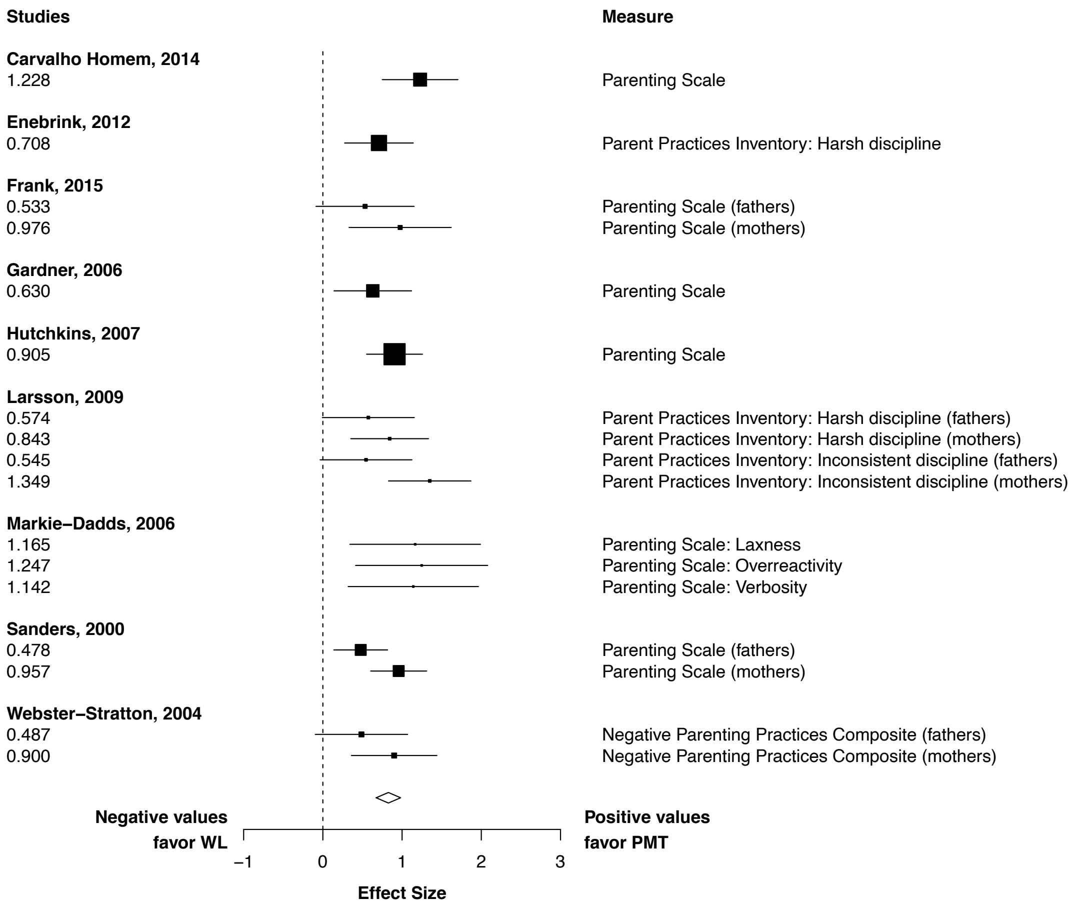

Note. The robust variance estimation of this data can be found in the fourth row of Figure 2.

Figure S5

Forest plot of effect sizes making up the analysis of parents' assessment of parental stress for standard PMT compared to WL at post-treatment

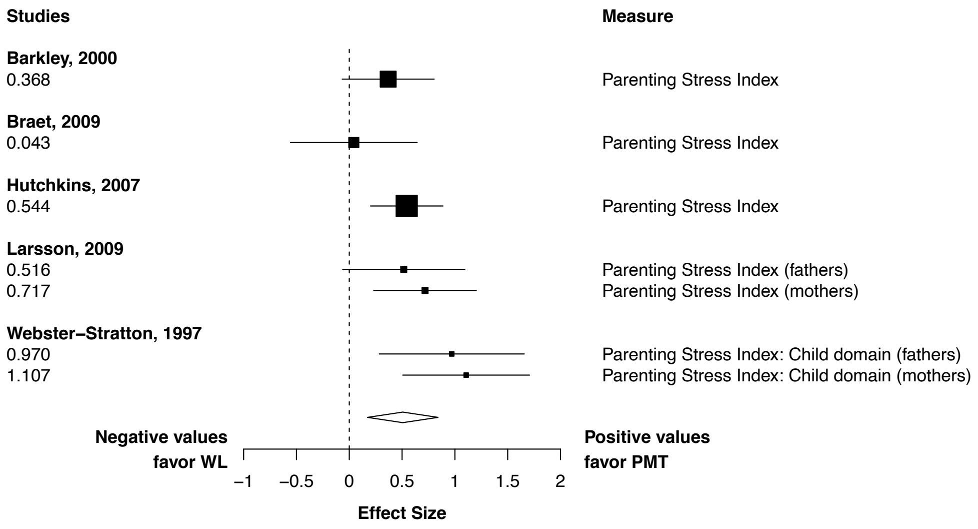

Note. The robust variance estimation of this data can be found in the fifth row of Figure 2.

Figure S6

Forest plot of effect sizes making up the analysis of parents' assessment of parental sense of competence for standard PMT compared to WL at post-treatment

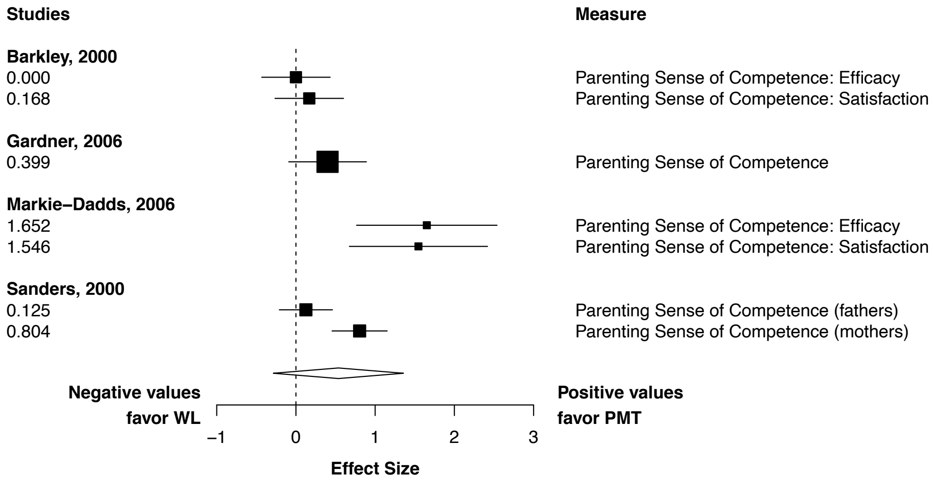

Note. The robust variance estimation of this data can be found in the sixth row of Figure 2.

Figure S7

Forest plot of effect sizes making up the analysis of clinicians' assessment of disruptive behavior for standard PMT compared to WL at post-treatment

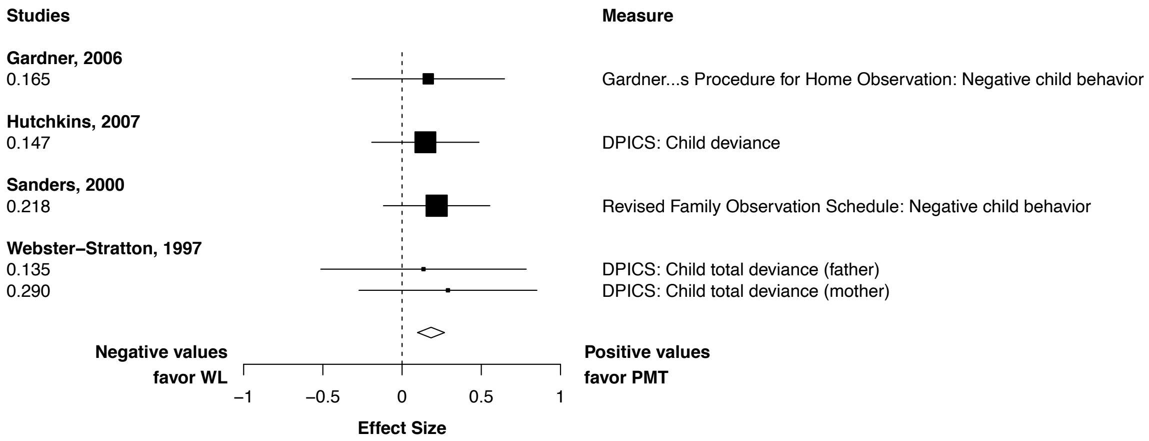

Note. The robust variance estimation of this data can be found in the seventh row of Figure 2.

Figure S8

Forest plot of effect sizes making up the analysis of clinicians' assessment of positive parenting skills for standard PMT compared to WL at post-treatment

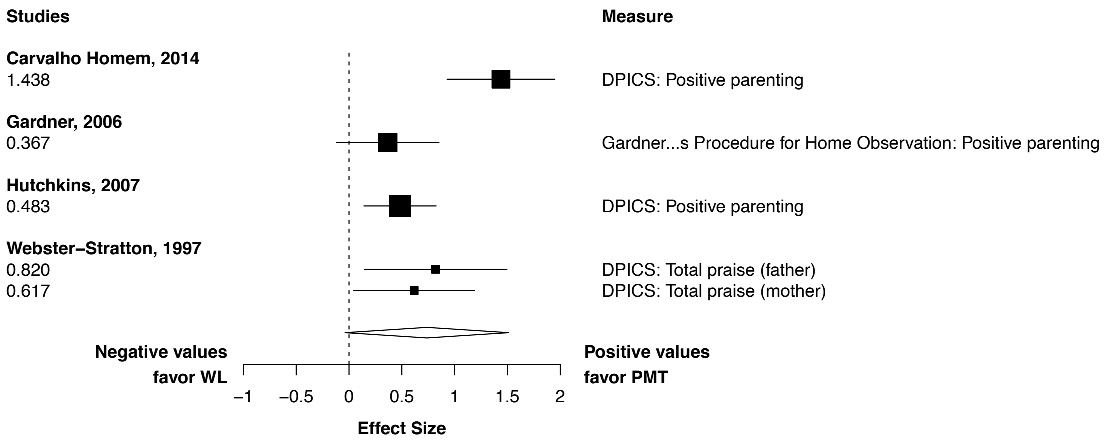

Note. The robust variance estimation of this data can be found in the ninth row of Figure 2.

Figure S9

Forest plot of effect sizes making up the analysis of clinicians' assessment of negative parenting skills for standard PMT compared to WL at post-treatment

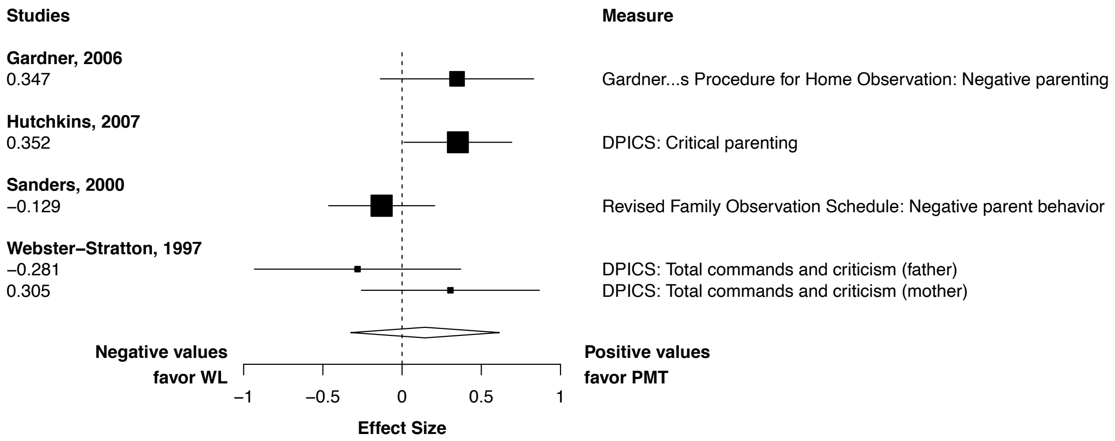

Note. The robust variance estimation of this data can be found in the tenth row of Figure 2.

Figure S10

Forest plot of effect sizes making up the analysis of teachers' assessment of disruptive behavior for standard PMT compared to WL at post-treatment

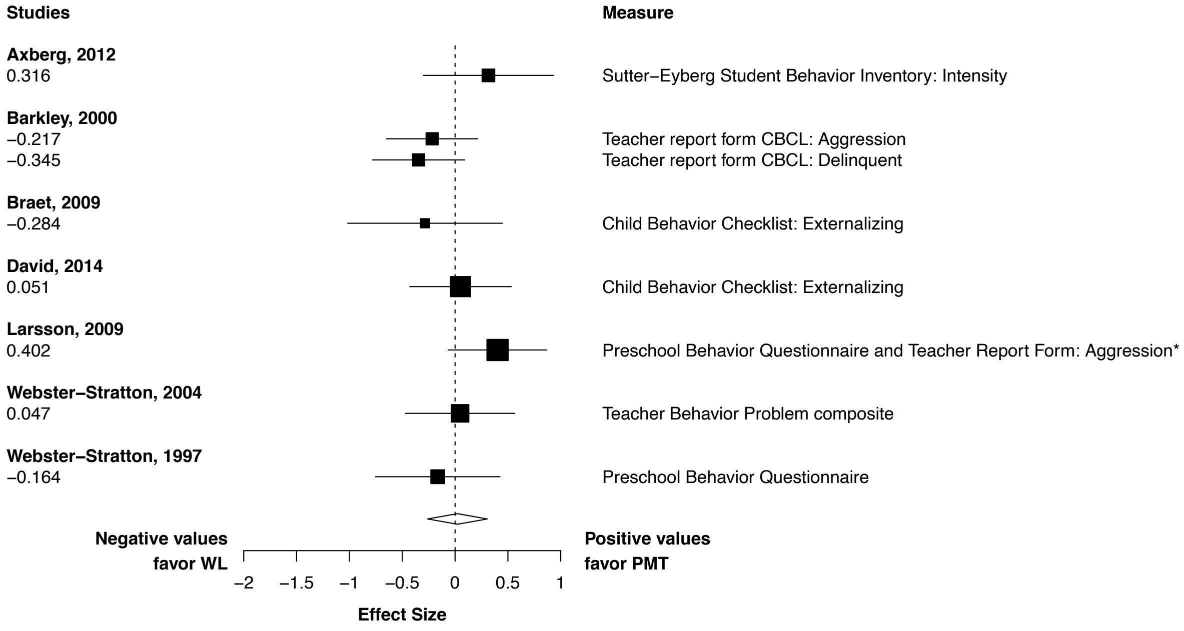

Note. The robust variance estimation of this data can be found in the eleventh row of Figure 2. * (Larsson, 2009) = Data from Drugli et al., 2006.

Figure S11

Forest plot of effect sizes making up the analysis of parents' assessment of disruptive behavior for PCIT compared to WL at post-treatment

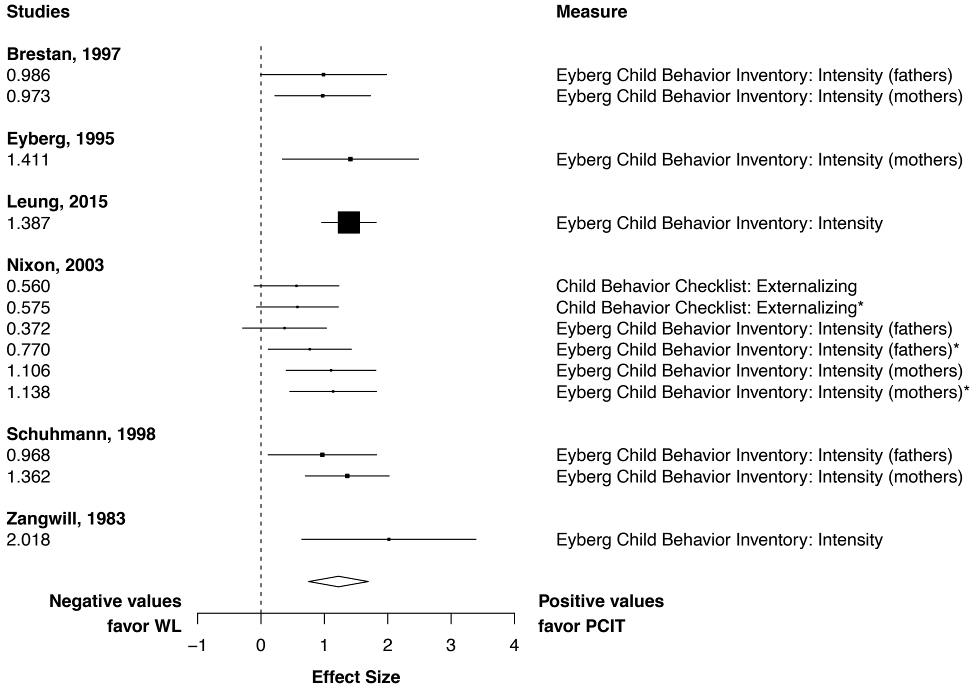

Note. The robust variance estimation of this data can be found in the first row of Figure 3. * = Abbreviated PCIT.

Figure S12

Forest plot of effect sizes making up the analysis of parents' assessment of parental stress for PCIT compared to WL at post-treatment

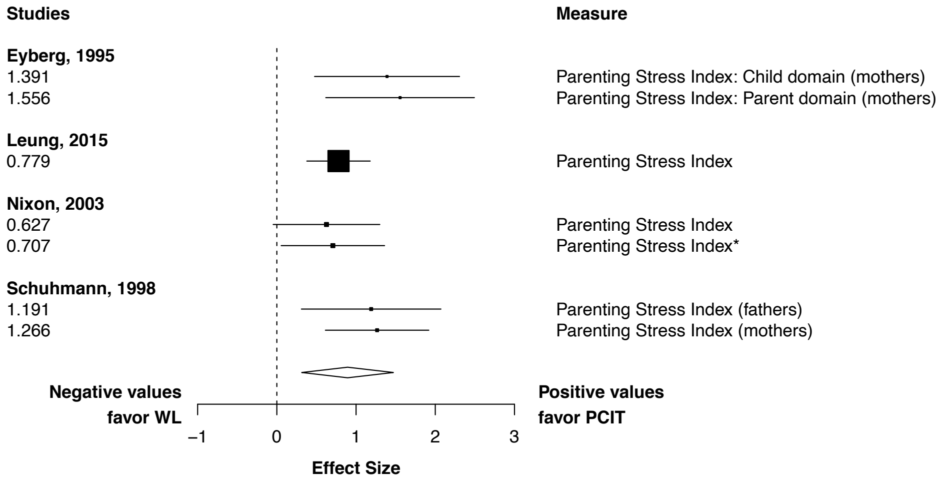

Note. The robust variance estimation of this data can be found in the fifth row of Figure 3. * = Abbreviated PCIT.

Figure S13

Forest plot of effect sizes making up the analysis of clinicians' assessment of positive parental strategies for PCIT compared to WL at post-treatment

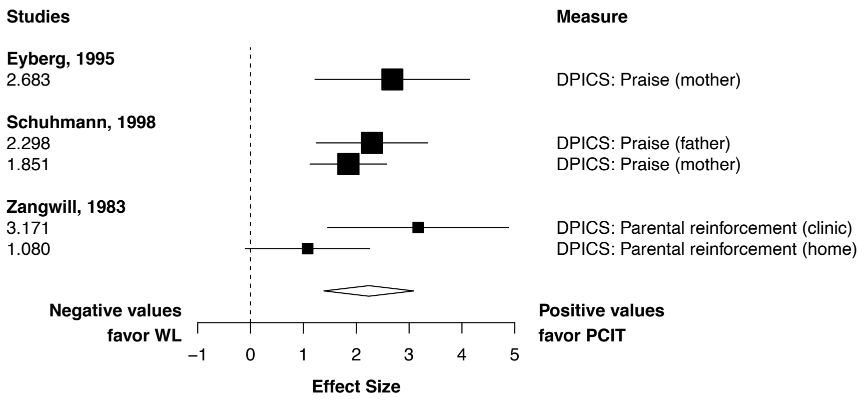

Note. The robust variance estimation of this data can be found in the seventh row of Figure 3.

Figure S14

Forest plot of effect sizes making up the analysis of clinicians' assessment of negative parental strategies for PCIT compared to WL at post-treatment

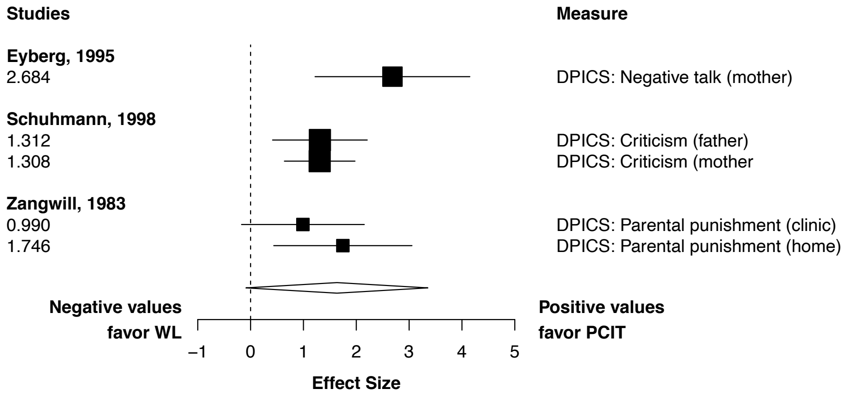

Note. The robust variance estimation of this data can be found in the eight row of Figure 3.

Figure S15

Forest plot of effect sizes making up the analysis of parents' assessment of disruptive behavior for PMT with child CBT compared to WL at post-treatment

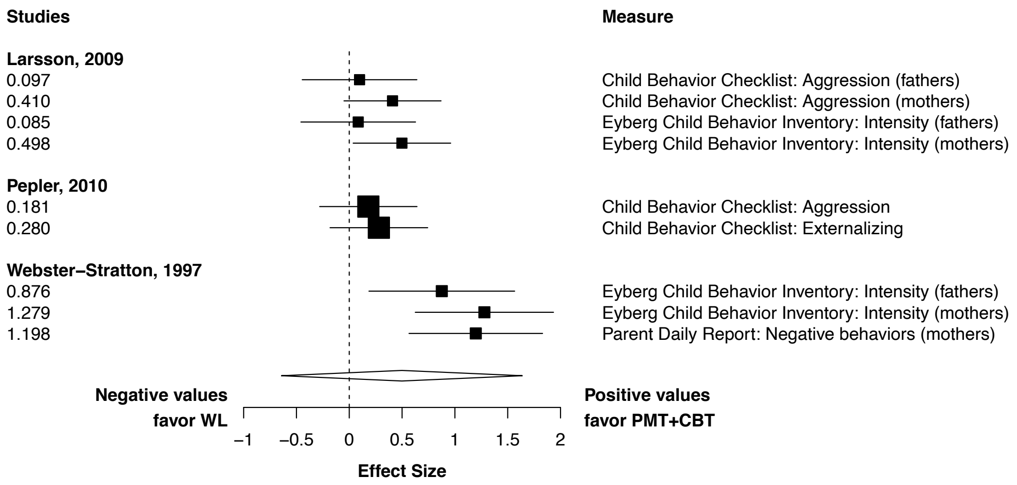

Note. The robust variance estimation of this data can be found in the ninth row of Figure 3.

Figure S16

Forest plot of effect sizes making up the analysis of teachers' assessment of disruptive behavior for PMT with child CBT compared to WL at post-treatment

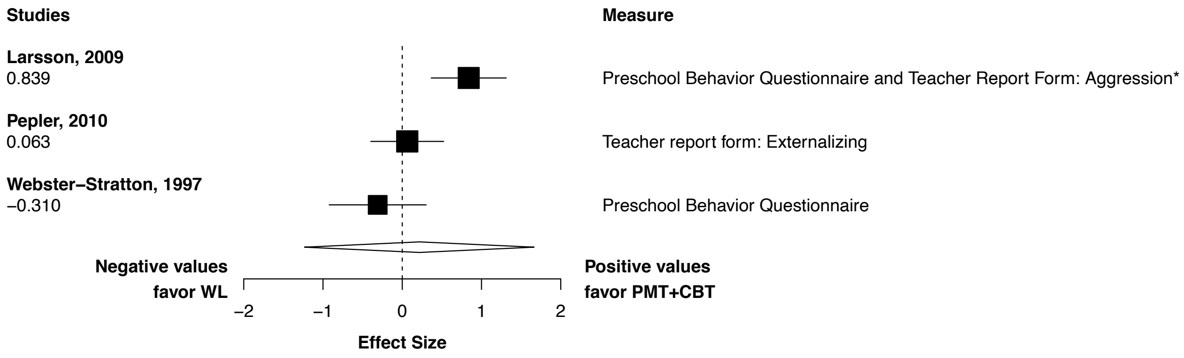

Note. The robust variance estimation of this data can be found in the fourteenth row of Figure 3. * = Data from Drugli et al., 2006.

Figure S17

Forest plot of effect sizes making up the analysis of parents' assessment of disruptive behavior for standard PMT compared to PMT with child CBT at post-treatment

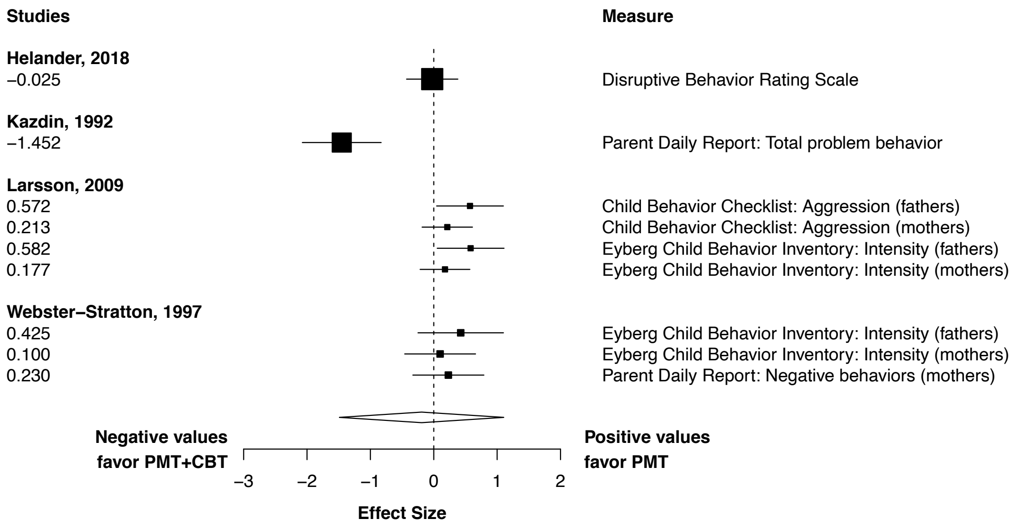

Note. The robust variance estimation of this data can be found in the first row of Figure 5.

Figure S18

Forest plot of effect sizes making up the analysis of parents' assessment of social skills for standard PMT compared to PMT with child CBT at post-treatment

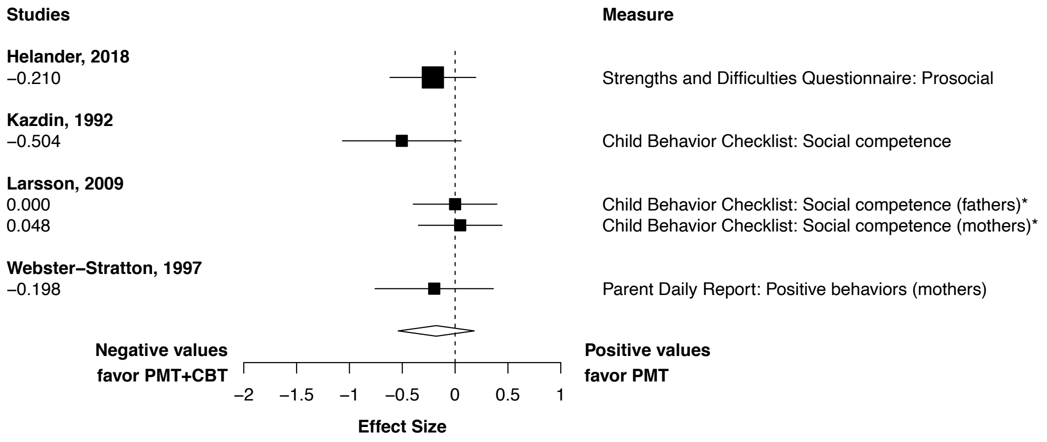

Note. The robust variance estimation of this data can be found in the second row of Figure 5. * = Data from Drugli et al., 2007.

Figure S19

Forest plot of effect sizes making up the analysis of parents' assessment of parental stress for standard PMT compared to PMT with child CBT at post-treatment

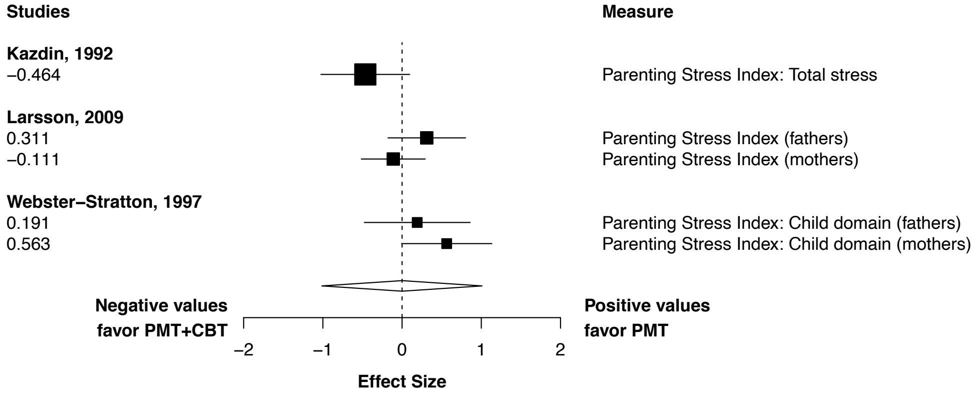

Note. The robust variance estimation of this data can be found in the fifth row of Figure 5.

Figure S20

Forest plot of effect sizes making up the analysis of parents' assessment of disruptive behavior for standard PMT compared to PMT with child CBT 12 months after treatment

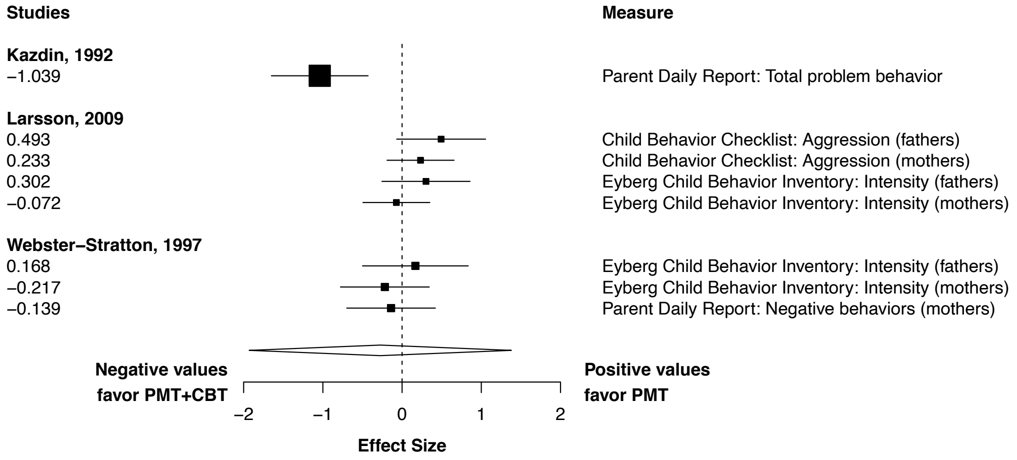

Note. The robust variance estimation of this data can be found in the sixth row of Figure 5.

Figure S21

Forest plot of effect sizes making up the analysis of parents' assessment of social skills for standard PMT compared to PMT with child CBT 12 months after treatment

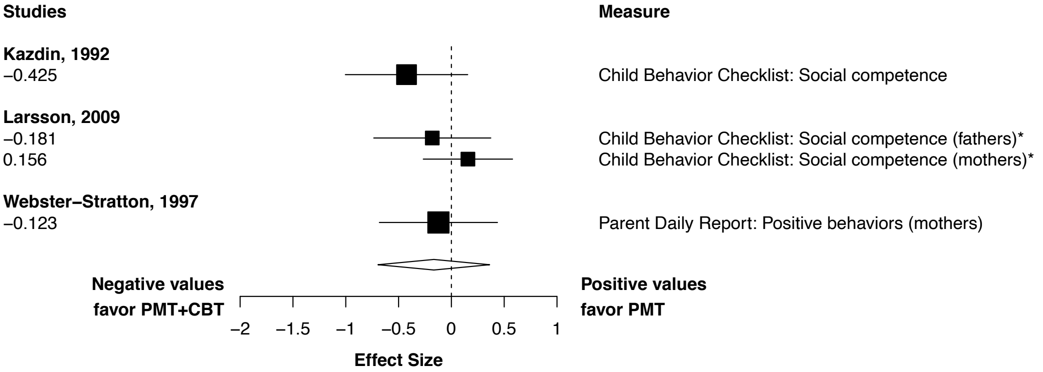

Note. The robust variance estimation of this data can be found in the seventh row of Figure 5. * = Data from Drugli et al., 2007.

Figure S22

Forest plot of effect sizes making up the analysis of parents' assessment of parental stress for standard PMT compared to PMT with child CBT 12 months after treatment

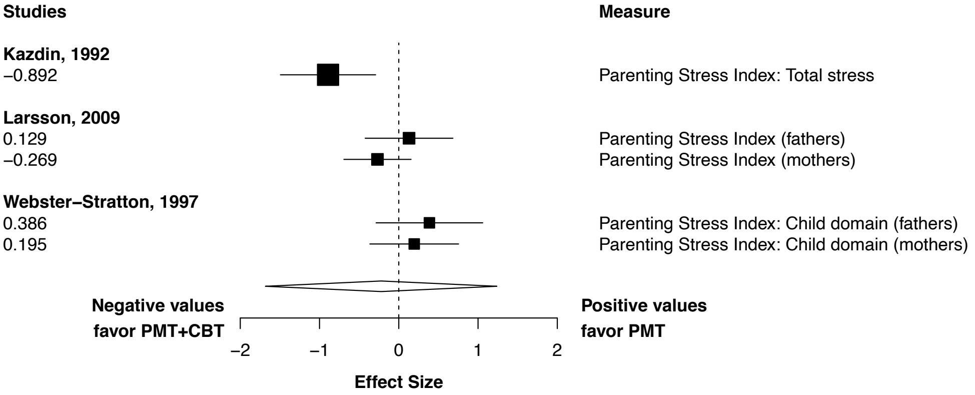

Note. The robust variance estimation of this data can be found in the tenth row of Figure 5.
