# Supplementary material for: The Efficacy of Parent Management Training With or Without Involving the Child in the Treatment Among Children with Clinical Levels of Disruptive Behavior: A Meta-analysis
Source: Child Psychiatry Hum Dev. 2022 Jul 5;55(1):164–81. doi: 10.1007/s10578-022-01367-y (PMC10796477; doi:10.1007/s10578-022-01367-y)
Supplement: Supplementary file 2 — Supplementary file2 (DOCX 2054 KB) [file 10578_2022_1367_MOESM2_ESM.docx]

| **Table S1**  *Descriptive data of baseline scores for the Eyberg Child Behavior Inventory Intensity Subscale for studies, divided by type of comparison and inclusion criteria* | | | | | |
| --- | --- | --- | --- | --- | --- |
| Condition 1 | Condition 2 | Inclusion criteria (*k*) | Combined  Mean (SD) | Condition 1  Mean (SD) | Condition 2  Mean (SD) |
| PMT | WL |  |  |  |  |
|  |  | ODD (2) | 154 (26) | 155 (24) | 154 (28) |
|  |  | Cut-off (8) | 150 (27) | 150 (27) | 149 (27) |
|  |  | Total (10) | 151 (27) | 151 (27) | 150 (28) |
| PCIT | WL |  |  |  |  |
|  |  | ODD (3) | 170 (28) | 167 (25) | 173 (32) |
|  |  | Cut-off (3) | 166 (21) | 168 (23) | 164 (19) |
|  |  | Total (6) | 168 (25) | 168 (24) | 167 (25) |
| PMT + Child CBT | WL |  |  |  |  |
|  |  | ODD (2) | 153 (27) | 153 (26) | 154 (28) |
|  |  | Cut-off (0) | - | - | - |
|  |  | Total (3) | 153 (27) | 153 (26) | 154 (28) |
| PMT + Child CBT | PMT |  |  |  |  |
|  |  | ODD (2) | 154 (25) | 153 (26) | 155 (24) |
|  |  | Cut-off (0) | - | - | - |
|  |  | Total (2) | 154 (25) | 153 (26) | 155 (24) |

*Note.* Means and standard deviations are derived from sample means and sample standard deviations reported in each study using the sample.decomp function from the R package Utilities (O'Neill, 2021. utilities: Data Utility Functions. R package version 0.3.0. https://CRAN.R-project.org/package=utilities). Whenever possible, numbers are based on the scores of both mothers and fathers. However, sometimes only one of these scores are given. The Combined column is derived by combining Condition 1 and Condition 2. A robust variance estimation moderator analysis with PMT vs. WL as one level and PCIT vs. WL as the other level could not show a difference between the estimates (p=0.90). k = Number of studies; PMT = Parent Management Training; PCIT = Parent Child Interaction Therapy; PMT + child CBT = PMT with child CBT, WL = Waiting list; ODD = ODD diagnosis diagnosed by clinician using structured diagnostic assessment; Cut-off = Studies on children with disruptive behavior problems above clinical cut-off.

**Figure S1**

*Forest plot of effect sizes making up the analysis of parents’ assessment of disruptive behavior for standard PMT compared to WL at post-treatment*

*Note*. The robust variance estimation of this data can be found in the first row of Figure 2.

**Figure S2**

*Forest plot of effect sizes making up the analysis of parents’ assessment of social skills for standard PMT compared to WL at post-treatment*

**

*Note*. The robust variance estimation of this data can be found in the second row of Figure 2. * = Data from Drugli et al., 2007.

**Figure S3**

*Forest plot of effect sizes making up the analysis of parents’ assessment of positive parenting skills for standard PMT compared to WL at post-treatment*

*Note*. The robust variance estimation of this data can be found in the third row of Figure 2.

**Figure S4**

*Forest plot of effect sizes making up the analysis of parents’ assessment of negative parenting skills for standard PMT compared to WL at post-treatment*

*Note*. The robust variance estimation of this data can be found in the fourth row of Figure 2.

**Figure S5**

*Forest plot of effect sizes making up the analysis of parents’ assessment of parental stress for standard PMT compared to WL at post-treatment*

*Note*. The robust variance estimation of this data can be found in the fifth row of Figure 2.

**Figure S6**

*Forest plot of effect sizes making up the analysis of parents’ assessment of parental sense of competence for standard PMT compared to WL at post-treatment*

*Note*. The robust variance estimation of this data can be found in the sixth row of Figure 2.

**Figure S7**

*Forest plot of effect sizes making up the analysis of clinicians’ assessment of disruptive behavior for standard PMT compared to WL at post-treatment*

*Note*. The robust variance estimation of this data can be found in the seventh row of Figure 2.

**Figure S8**

*Forest plot of effect sizes making up the analysis of clinicians’ assessment of positive parenting skills for standard PMT compared to WL at post-treatment*

*Note*. The robust variance estimation of this data can be found in the ninth row of Figure 2.

**Figure S9**

*Forest plot of effect sizes making up the analysis of clinicians’ assessment of negative parenting skills for standard PMT compared to WL at post-treatment*

**

*Note*. The robust variance estimation of this data can be found in the tenth row of Figure 2.

**Figure S10**

*Forest plot of effect sizes making up the analysis of teachers’ assessment of disruptive behavior for standard PMT compared to WL at post-treatment*

*Note*. The robust variance estimation of this data can be found in the eleventh row of Figure 2. * (Larsson, 2009) = Data from Drugli et al., 2006.

**Figure S11**

*Forest plot of effect sizes making up the analysis of parents’ assessment of disruptive behavior for PCIT compared to WL at post-treatment*

*Note*. The robust variance estimation of this data can be found in the first row of Figure 3. * = Abbreviated PCIT.

**Figure S12**

*Forest plot of effect sizes making up the analysis of parents’ assessment of parental stress for PCIT compared to WL at post-treatment*

*Note*. The robust variance estimation of this data can be found in the fifth row of Figure 3. * = Abbreviated PCIT.

**Figure S13**

*Forest plot of effect sizes making up the analysis of clinicians’ assessment of positive parental strategies for PCIT compared to WL at post-treatment*

**

*Note*. The robust variance estimation of this data can be found in the seventh row of Figure 3. * = Abbreviated PCIT.

**Figure S14**

*Forest plot of effect sizes making up the analysis of clinicians’ assessment of negative parental strategies for PCIT compared to WL at post-treatment*

**

*Note*. The robust variance estimation of this data can be found in the eight row of Figure 3. * = Abbreviated PCIT.

**Figure S15**

*Forest plot of effect sizes making up the analysis of parents’ assessment of disruptive behavior for PMT with child CBT compared to WL at post-treatment*

*Note*. The robust variance estimation of this data can be found in the ninth row of Figure 3.

**Figure S16**

*Forest plot of effect sizes making up the analysis of teachers’ assessment of disruptive behavior for PMT with child CBT compared to WL at post-treatment*

*Note*. The robust variance estimation of this data can be found in the fourteenth row of Figure 3. * = Data from Drugli et al., 2006.

**Figure S17**

*Forest plot of effect sizes making up the analysis of parents’ assessment of disruptive behavior for standard PMT compared to PMT with child CBT at post-treatment*

*Note*. The robust variance estimation of this data can be found in the first row of Figure 5.

**Figure S18**

*Forest plot of effect sizes making up the analysis of parents’ assessment of social skills for standard PMT compared to PMT with child CBT at post-treatment*

*Note*. The robust variance estimation of this data can be found in the second row of Figure 5. * = Data from Drugli et al., 2007.

**Figure S19**

*Forest plot of effect sizes making up the analysis of parents’ assessment of parental stress for standard PMT compared to PMT with child CBT at post-treatment*

*Note*. The robust variance estimation of this data can be found in the fifth row of Figure 5.

**Figure S20**

*Forest plot of effect sizes making up the analysis of parents’ assessment of disruptive behavior for standard PMT compared to PMT with child CBT 12 months after treatment*

*Note*. The robust variance estimation of this data can be found in the sixth row of Figure 5.

**Figure S21**

*Forest plot of effect sizes making up the analysis of parents’ assessment of social skills for standard PMT compared to PMT with child CBT 12 months after treatment*

*Note*. The robust variance estimation of this data can be found in the seventh row of Figure 5. * = Data from Drugli et al., 2007.

**Figure S22**

*Forest plot of effect sizes making up the analysis of parents’ assessment of parental stress for standard PMT compared to PMT with child CBT 12 months after treatment*

*Note*. The robust variance estimation of this data can be found in the tenth row of Figure 5.
